# Economic Impact of Dengue: Multicenter Study across Four Brazilian Regions

**DOI:** 10.1371/journal.pntd.0004042

**Published:** 2015-09-24

**Authors:** Celina Maria Turchi Martelli, Joao Bosco Siqueira, Mirian Perpetua Palha Dias Parente, Ana Laura de Sene Amancio Zara, Consuelo Silva Oliveira, Cynthia Braga, Fabiano Geraldo Pimenta, Fanny Cortes, Juan Guillermo Lopez, Luciana Ribeiro Bahia, Marcia Costa Ooteman Mendes, Michelle Quarti Machado da Rosa, Noemia Teixeira de Siqueira Filha, Dagna Constenla, Wayner Vieira de Souza

**Affiliations:** 1 Department of Public Health, Aggeu Magalhaes Research Centre, Oswaldo Cruz Foundation, Recife, Brazil; 2 Department of Community Health, Institute of Tropical Pathology and Public Health, Federal University of Goias, Goiania, Brazil; 3 Department of Community Health, Universidade Estadual do Piaui, Teresina, Brazil; 4 Universidade do Estado do Para, Belem, Brazil; 5 Department of Health Surveillance, Secretaria Municipal Saude Belo Horizonte, Belo Horizonte, Brazil; 6 Department of Health Science, Universidade de Pernambuco, Recife, Brazil; 7 Health Economics and Market Access, Sanofi Pasteur Latin America, Mexico City, Mexico; 8 Department of Internal Medicine, State University of Rio de Janeiro, Rio de Janeiro, Brazil; 9 Department of Global Health and Development, London School of Hygiene and Tropical Medicine, London, United Kingdom; 10 Department of International Health, Bloomberg School of Public Health, Johns Hopkins University, Baltimore, Maryland, United States of America; Oswaldo Cruz Foundation, BRAZIL

## Abstract

**Background:**

Dengue is an increasing public health concern in Brazil. There is a need for an updated evaluation of the economic impact of dengue within the country. We undertook this multicenter study to evaluate the economic burden of dengue in Brazil.

**Methods:**

We estimated the economic burden of dengue in Brazil for the years 2009 to 2013 and for the epidemic season of August 2012- September 2013. We conducted a multicenter cohort study across four endemic regions: Midwest, Goiania; Southeast, Belo Horizonte and Rio de Janeiro; Northeast: Teresina and Recife; and the North, Belem. Ambulatory or hospitalized cases with suspected or laboratory-confirmed dengue treated in both the private and public sectors were recruited. Interviews were scheduled for the convalescent period to ascertain characteristics of the dengue episode, date of first symptoms/signs and recovery, use of medical services, work/school absence, household spending (out-of-pocket expense) and income lost using a questionnaire developed for a previous cost study. We also extracted data from the patients’ medical records for hospitalized cases. Overall costs per case and cumulative costs were calculated from the public payer and societal perspectives. National cost estimations took into account cases reported in the official notification system (SINAN) with adjustment for underreporting of cases. We applied a probabilistic sensitivity analysis using Monte Carlo simulations with 90% certainty levels (CL).

**Results:**

We screened 2,223 cases, of which 2,035 (91.5%) symptomatic dengue cases were included in our study. The estimated cost for dengue for the epidemic season (2012–2013) in the societal perspective was US$ 468 million (90% CL: 349–590) or US$ 1,212 million (90% CL: 904–1,526) after adjusting for under-reporting. Considering the time series of dengue (2009–2013) the estimated cost of dengue varied from US$ 371 million (2009) to US$ 1,228 million (2013).

**Conclusions:**

The economic burden associated with dengue in Brazil is substantial with large variations in reported cases and consequently costs reflecting the dynamic of dengue transmission.

## Author Summary

The World Health Organization estimates that 50–100 million dengue infections occur annually. However, estimates based on cartographic modeling approaches suggest that up to 390 million dengue infections annually. Dengue has become an increasing public health concern in the Americas. A substantial number of cases in the Americas are reported in Brazil. There is a need for an updated evaluation of the economic impact of dengue in Brazil so as to allow decision/policy makers to make informed decisions on resource allocation and strategic planning. We estimated the economic burden of dengue in four regions. National cost estimates were based on the number of cases reported in Sistema de Informacao de Agravos de Notificacao (SINAN). Overall, 2,035 (91.5%) dengue cases were recruited to our study. More than 2 million suspected dengue cases and 569 deaths were reported in SINAN from September 2012 to August 2013. The annual national economic burden was US$ 164 million from the public payer perspective, but may be as high as US$ 447 million (adjusting for underreporting). From the societal perspective, the economic burden was US$ 468 million, but may be as high as US$ 1,212 million. The economic burden associated with dengue in Brazil is substantial.

## Introduction

Dengue is a viral infection transmitted by *Aedes* mosquitoes, with global distribution, mainly in the tropical regions [1]. Infection with one of the four antigenically distinct dengue serotypes (serotypes 1, 2, 3, and 4) is often asymptomatic/inapparent or mildly symptomatic, but has the potential to escalate to dengue fever and subsequently, to life-threatening dengue hemorrhagic fever or dengue shock syndrome, and death. Although life-long immunity to the infecting serotype may develop, the more severe or life-threatening cases of dengue are more often associated with subsequent secondary infection by heterologous dengue serotypes [1].

Contemporary global estimates from the World Health Organization suggest that 50–100 million dengue infections occur annually [2].A more recent estimate, based on cartographic modeling approaches and data from various published sources between 1960 and 2012, suggests that there are about 390 million dengue infections per year with 96 million apparent/symptomatic cases of the disease [3]. Although the majority of dengue infections occur in Asia [3], there has been a dramatic increase in the number of reported dengue cases in the Americas over the last decade [4–6]. Over 50 million dengue infections were estimated (using cartographic modeling approaches)in the Americas in 2010, and of these, about 40%(21.8 million infections) occurred in Brazil [3]. Recent surveillance data from the Brazilian national notifiable diseases information system (SINAN; Sistema de Informacao de Agravos de Notificacao) showed that there were more than 2 million dengue cases reported in 2013, the highest annual incidence registered in Brazil since dengue surveillance was implemented in the 1980´s [7]. The increase in the incidence of dengue was probably due to the introduction of dengue serotype-4 along with the rapid spread and co-circulation of the other serotypes (serotypes 1, 2, and 3). In addition, the reporting rates may have increased due to higher dengue awareness among the population and the health workers.

Dengue can impose a significant economic and humanistic burden in countries where the disease is endemic and, as such, estimating the associated economic and disease burden can help inform policy makers and assist them in setting priorities for disease-management strategies and for the introduction of new technologies [8]. To date, there has been one previous cost evaluation of the economic burden of dengue episodes in Brazil (undertaken only in the Midwest region, city of Goiania in 2005), which was part of an international study that included five countries in the Americas (Brazil, El Salvador, Guatemala, Panama, and Venezuela) [9], and was subsequently updated as part of a later study [10]. These previous evaluations, however, estimated the cost of dengue cases without taking into account seasonal fluctuation or costs associated with dengue outbreaks. Nonetheless, the estimated cost of dengue illness across the Americas between 2000 and 2007 was substantial, at US$2.1 billion per year (2010 US$), with the majority of costs associated with ambulatory cases rather than hospitalized cases and with substantial year to year variation [10]. Brazil accounted for about 40% (US$878.2 million) of the total costs in the Americas. Since these publications, recommendations and guidelines have been developed for estimating the burden and socio-economic costs of dengue [11–13]. There is marked variation in dengue transmission across time and by serotype circulation, as previously published [14–18]. As such, there is a need for an updated evaluation of the economic impact of dengue in Brazil that also includes more regions within the country and adheres to the new guidelines and recommendations.

The Brazilian health system is a complex mix of both public and private sectors [19, 20]. A cost evaluation needs to consider the perspective of both sectors [12]. The aim of this study was to evaluate the economic burden of dengue from the public payer and societal perspectives across four endemic regions in Brazil, and to extrapolate these costs to the whole country.

## Methods

### Study design

This was a prospective, multicenter, observational study aimed at measuring the direct and indirect costs associated with dengue illness across six sites in four endemic regions of Brazil. The time horizon of analysis was one year, from September 2012 to August 2013, which takes account of dengue seasonality.

### Ethical considerations

The protocol was approved by each centers’ Ethical Committee and registered at the Brazilian Ethical Office (PlataformaBrasil: http://aplicacao.saude.gov.br/plataformabrasil) under number: 94.121/2012. All participants, or their parents/guardians (for minors aged <18 years), provided written informed consent before study entry.

### Study setting

**Fig 1:**
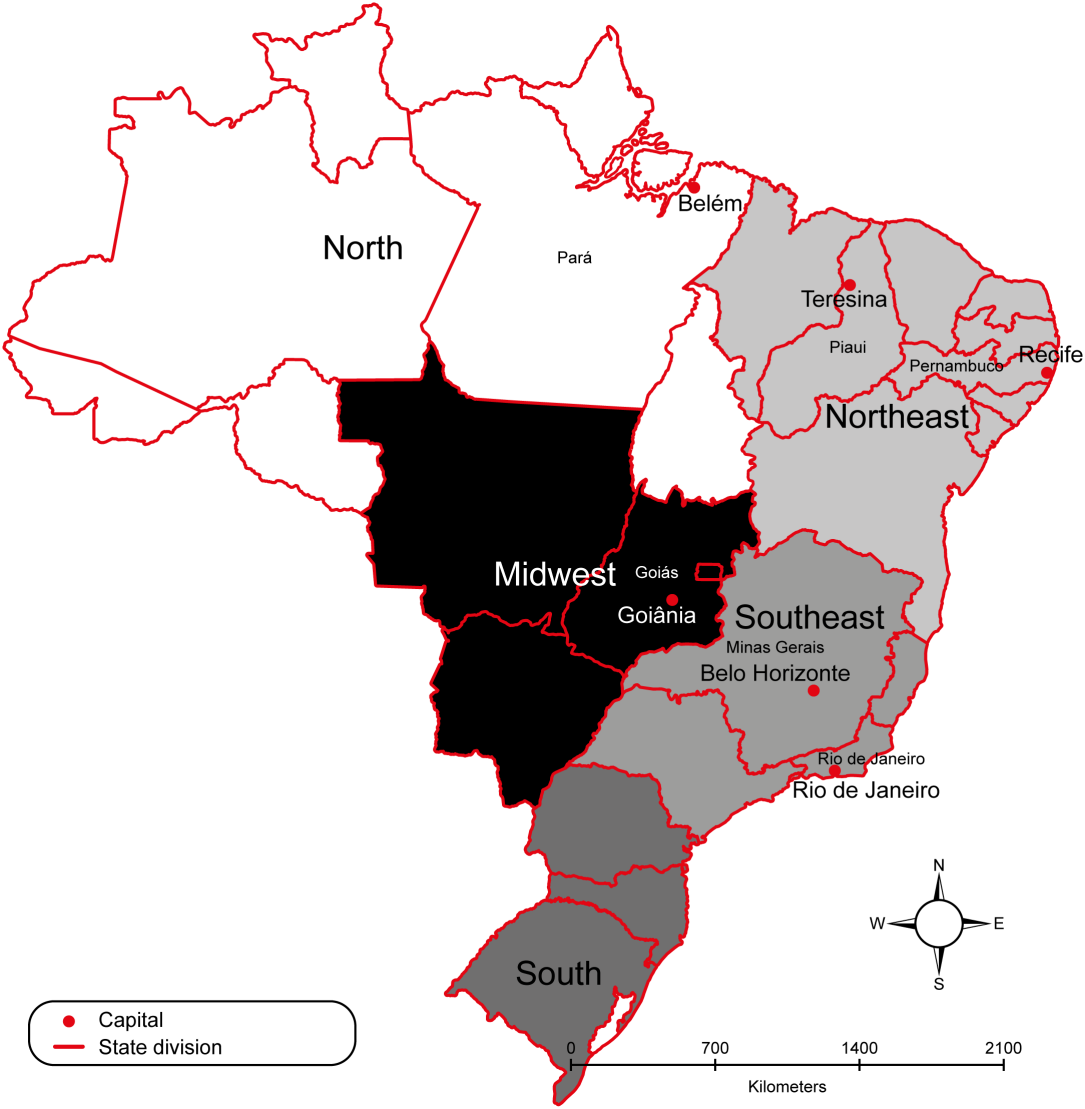
Map of Brazil: Regions and study sites.

We selected six state capitals located in four endemic regions in Brazil: (1) Midwest: Goiania/Goias State, (2) Southeast: Belo Horizonte/Minas Gerais State and Rio de Janeiro/Rio de Janeiro State, (3) Northeast: Teresina/Piaui State, Recife/Pernambuco State and (4) North: Belem/Para State (Fig 1). We selected capital cities located in regions of high transmission of dengue infection in the last decade [6]. Capital cities were chosen because they are densely populated areas with the capacity for research development and health staff to perform the fieldwork in a timely manner. We did not include cities in the Southern region as dengue transmission was only concentrated in one area of Parana State.

### Study population

The study population consisted of patients with clinically suspected dengue. Clinically suspected dengue was defined in accordance with the Brazilian dengue guidelines as fever plus two or more of the following manifestations: headache, retro-orbital pain, myalgia, arthralgia, prostration or rash (with or without the presence of hemorrhage) [21]. All sites included children (aged <15 years) and adults. Residents from other municipalities, individuals unable to respond to an interview request, those who refused to give informed consent and those whose residential address could not be located/accessed were excluded from the study. In five of the six sites, the fieldwork was conducted in 2013.The other site, Teresina (Northeast), the fieldwork was conducted during 2012 and 2013.

### Sample size

We estimated a sample size of 300 ambulatory patients and 100 hospitalized patients would be required for each site in the original protocol. This estimate was based on the assumption that these numbers were sufficient to sample patients of all age groups and disease severity, and to have sufficient statistical robustness. A minimum of approximately 30 hospitalized patients were to be recruited at the sites with low virus circulation.

### Interviews

We contacted all patients with clinically suspected dengue or their care-givers so as to interview them with regard to their illness. Interviews with the patients or their care-givers were planned for two phases.

#### Phase 1. Screening interviews

ambulatory and hospitalized cases (ambulatory cases were defined those cases who attended healthcare facilities, and hospitalized as those cases who required an hospital stay of 24 hours or more). This screening interview was undertaken to recruit patients or their care-givers, to explain project objectives, to obtain informed consent, to collect baseline information and schedule household interviews. We arranged the household interview 15–20 days after the onset of clinical symptoms.

#### Phase 2. Household interviews

Cases identified during phase 1 or their care-givers were interviewed using a questionnaire (described below) after resolution of their symptoms. Field-workers performed the interviews using Tablet devices and entered the answers directly whenever possible.

### Data collection and sources

We adopted a questionnaire for use during interviews with the patients or their care-givers identified, which was developed in a previous multi-country cost study [9], to collect resource use data in public and private ambulatory clinics and hospitals (see S1 Text). This questionnaire helped document: the characteristics of the dengue episode; history of previous dengue illness; date of initial appearance of symptoms/signs and their resolution; use of medical services(medical visits; medications, laboratory exams); work and school absences; household spending (out-of-pocket expenses); and income lost (loss of productivity). We augmented the questionnaire used in the previous study by adding questions about medication use, whether dengue was laboratory confirmed, and about laboratory tests and x-ray examinations undertaken. A pilot study was conducted to evaluate the feasibility of the questionnaire and the field strategy before the full investigation. In addition to the interviews, we extracted data from the patients’ medical records for those who were hospitalized to gather resource use information (hospital stay, laboratory/exam and medication). The information collected regarding resource is subject for an accompanying paper.

We used the ABEP classification (Criterio de Classificacao Economica Brasil, CCEB 2012, available at www.abep.org) to determine the economic level of the patients or their care-givers. This classification estimates the purchasing power of individuals and families. This index is equivalent to the mean family income per month with 8 economic classes as follows: top level of income [A1 (US$ 5,698); A2 (US$ 3,711)]; mid-level income [B1 (US$ 1,947); B2 (US$ 1,131)]; low-level income [C1 (US$ 679); C2 (US$ 451)]; very low-level income [D (US$ 315) to E (US$210)].

### National surveillance system

According to the SINAN database, there were 2,013,274 suspected dengue cases reported between September 2012 and August 2013 (Table 1). The majority of these reported dengue cases were aged 15 years or older (approximately 1.7 million cases), of which, more than 50% of cases were in females. The majority (99.5%) of the reported cases were classified as “classical” dengue, 0.5% were classified as severe dengue cases (dengue with complications, dengue hemorrhagic fever and dengue shock syndrome). All four dengue serotypes co-circulated in Brazil, with serotype 1 and serotype 4 being the main serotypes detected during the study period. There were 569 deaths due to dengue disease reported in SINAN. Dengue epidemics were reported in two of the study regions during our investigations: the Southeast and Midwest neighboring regions with the cities of Belo Horizonte and Goiania registering dengue incidence rates ≥50 per 1,000 inhabitants (Table 1). The official definition of dengue epidemic by the national surveillance system is when the number of cases exceeds the number of cases of the upper limit relative to the incidence represented by diagram of control in a specific region [22]. It should be noted that SINAN is a passive surveillance system and it is likely that the number of cases would have been under-reported, particularly for non-hospitalized cases, as is the tendency with such surveillance systems [23].

**Table 1:** Dengue epidemiology in Brazil overall, and by site in the four regions (surveillance data from the Brazilian national notifiable diseases information system [SINAN] September 2012 to August 2013).

Variables	Brazil	Midwest	Southeast		Northeast		North
		Goiania	Belo Horizonte	Rio de Janeiro	Teresina	Recife	Belem
**Population (millions)** ^**a**^	190.733	1.2	2.4	6.3	1.2	1.5	1.4
**Reported cases** ^**b**^	2,013,274	60,076	132,614	74,116	2,694	4,073	1,763
**Female**	1,134,695(56.4%)	33,070	79,323	40,317	1,392	2,171	965
**Age groups (years)**							
**0–4**	68,562 (3.4%)	2,036	2,830	2,361	170	211	116
**5–14**	246,575 (12.2%)	6,135	14,594	9,372	413	557	267
**≥15**	1,684,472 (83.7%)	51,574	114,468	61,944	2,090	3,289	1,368
**Severe dengue cases** ^**c**^	7,664 (0.5%)	844	67	240	5	14	8
**Fatal cases**	569	20	9	24	2	3	1
**Incidence (per 1,000)**	10.6	50.1	55.3	11.8	2.2	2.7	1.3

^a^Censo Demografico 2010 [24], Instituto Brasileiro de Geografia e Estatistica (IBGE) [25];

^b^Reported cases: September 2012 –August 2013,Source, SINAN;

^c^Ministry of Health Severe Dengue Cases definition includes: Dengue With Complications, Dengue Hemorrhagic Fever and Dengue Shock Syndrome. Dengue cases classified does not add up the total reported cases.

### Brazilian healthcare system

The Brazilian health system consists of a complex public-private mix healthcare delivery/utilization and financing system [20]. The public sector comprises the Unified Health System (Sistema Unico de Saude—SUS) and is considered to be a universal system (open to all) funded by the government. Citizens have the right to receive preventive measures and treatment, free of charge under this system. The private sector offers health service by direct payment and/or covered by health insurance plans (Unimed; Plamta, MedPlan, Cassi and others). The participation of the private sector in the Brazilian health system is classified as “setor suplementar”/supplementary care [19].

### Cost of illness measurement

We used a micro-costing, bottom-up approach. The costs of ambulatory or hospitalized cases were calculated as the average cost of each component of direct and indirect cost as shown in S1 and S2 Tables. All costs were initially calculated in the local currency (Real) and converted to US dollars (US$) using an average exchange rate (R$ 1 = US$ 0.44079 on November 20, 2013) by OANDA (www.oanda.com).

The cost for a medical visit for an ambulatory case in the public sector included the SUS value for one medical visit to the emergency department (US$ 4.85) [26]. The inclusion of a medical visit to the emergency department, which is the highest price paid by SUS, was done because the current cost of an ambulatory visit was considered to be undervalued [27]. In the private sector, the unit cost for each medical visit funded from a health insurance plan was taken from the Unimed plan (US$ 26.45) since this was the insurer used by the majority of the study population [28]. The direct cost of hospitalized patients included the cost of hospitalization (hospital stay) and the cost of ambulatory care for those who sought ambulatory treatment before inpatient hospitalization. We calculated the percentage of hospitalized patients who received previous ambulatory care in each city and added these costs to the hospital costs. For both medication (http://portal.anvisa.gov.br) and laboratory tests (http://sigtap.datasus.gov.br) the public sector costs were taken from Brazilian Government data. Private sector medication costs were taken from Guia Farmaceutico Brasindice (January, 2013) and laboratory tests from the Associacao Medica Brasileira (http://www.amb.org.br/_arquivos/_downloads/cbhpm_2012.pdf).

We estimated the loss in productivity due to illness and death. The value of lost productivity due to illness took into account the number of work days lost by patients or caregivers during the course of illness. We also included the number of days of school missed by patients (see S1 and S2 Tables). The number of work days lost or school days missed were derived from the household interviews. Loss of income by patient or care-giver was based on the perceived the monetary value of the previous month’s income. The value of lost school or college days took into account the level of education in each state and was based on the annual cost per student of US$ 6,612.00 (http://www.jusbrasil.com.br/diarios/DOU/2012/11/19 and http://ultimosegundo.ig.com.br/educacao/educacao+basica+custa+mais+na+particular+superior+na+publica/n1597000724462.html).

### Data analysis

Data analyses were undertaken using SPSS 17.0, Microsoft Excel 2010 and Package R (accessed at: http://www.r-project.org/). The unit of analysis was a dengue episode, defined as a symptomatic dengue case with or without laboratory confirmation. We analyzed the patients with dengue by sites, stratified by whether they were ambulatory and hospitalized, in both public and private facilities. We carried out exploratory analysis for continuous variables to evaluate the distribution patterns and outliers. For skewed distributions, we used a non-parametric test (Kruskal Wallis test) to compare the number of days in hospital between sites.

Descriptive statistics, frequencies, means and standard deviation (SD) were calculated to compare costs across sites. We used the chi-square test to compare the distribution of the characteristics of dengue cases between sites and we used residual analysis to identify the significant heterogeneities among sites. The Tamhane post-hoc test was applied for multiple comparative analyses of means with non-homogenous variances. We opted to perform this first analytic approach using means and standard deviations (SD) as recommended for cost analyses [29].

To calculate the overall national cost we used the number of cases reported in Brazil and the number of cases reported in the six cities studied during the period (SINAN data). We adopted the ratio of ambulatory:hospital (9:1) as a parameter to estimate the number of ambulatory and hospitalized cases at the national level (Siqueira personal communication, 2013). We assumed that 65% of patients attended the public sector in the Southeast and Midwest regions and 75% in the North and Northeast regions [25]. The estimated cost per case was calculated as: the total amount of direct and indirect costs from the dengue episodes calculated in each of the six cities (primary data) divided by the number of estimated cases in the six cities of the study. We estimated the overall national cost by multiplying the estimated cost per case by the estimated number of ambulatory and hospitalized cases notified in Brazil between September 2012 and August 2013 (see S2 Text).

### Cost of fatal dengue cases

In our study, five deaths occurred during the study period, all at one site (Belo Horizonte). We estimated the cost of fatal cases at national level using the human capital approach to determine the cost associated with the loss of productivity from these cases [30]. We estimated the cost of fatal dengue as the lost income from premature death [31]. Based on the number of deaths in SINAN database (2011, 2012 and 2013), we estimated the average age at death due to dengue to be 45 years for males and 42 years for females. We calculated the years of life lost and used a standard discount rate of 3% per year. We estimated the remaining life labor expectancy at their age of death considering the minimum age of retirement for males of 65 years and 60 years for males and females, respectively. We multiplied the average discounted years by Brazil gross domestic product (GDP) per capita in 2012 (US$ 10,420.70).

### Sensitivity analysis

We applied a probabilistic sensitivity analysis to our data using RiskAMP software (Structured Data LLC 2005). We investigated how variations of ± 50% (min; max) affected the best estimate derived from our data by computing 10,000 Monte Carlo simulations. We used a beta-PERT distribution and reported the results in terms of certainty level (CL) bounds using 10 and 90 percentiles. We varied the mean values obtained by the field study (direct cost per case, total cost per case) and then estimated the annual costs of dengue at national level by ambulatory and hospital in both public payer and societal perspectives. Simulation of expansion factors took into account higher variability: EF used for ambulatory cases were 1.5; 3; and 6. We assumed the variation (1; 1.6; 2) for the EF for hospitalized cases. In the latter the simulation includes the EF = 1.6 cited in the literature [32].

### Extrapolating of dengue burden at national level

The estimated cost of dengue in Brazil was considered from two perspectives: public payer and societal. The public payer perspective included only direct costs in the public healthcare setting (medical visits, medications and laboratory/examinations). The societal perspective included direct and indirect costs, weighted by public and private sectors. Two scenarios were evaluated for each perspective (public payer and societal) as a sensitivity analysis to capture some of the uncertainty in the number of cases reported in SINAN. In the first scenario (base case), we used official registered dengue cases by SINAN to estimate the cost of dengue in Brazil (as described above). In the second scenario, we applied EFs yielded from sensitivity analysis: for ambulatory cases (EF = 3.2) and for hospitalized cases (EF = 1.6) in order to adjust for underreporting [32]. We have also extrapolated the dengue burden using temporal series from 2009 to 2013 (SINAN online database). We assumed the same parameters as described above.

## Results

**Fig 2:**
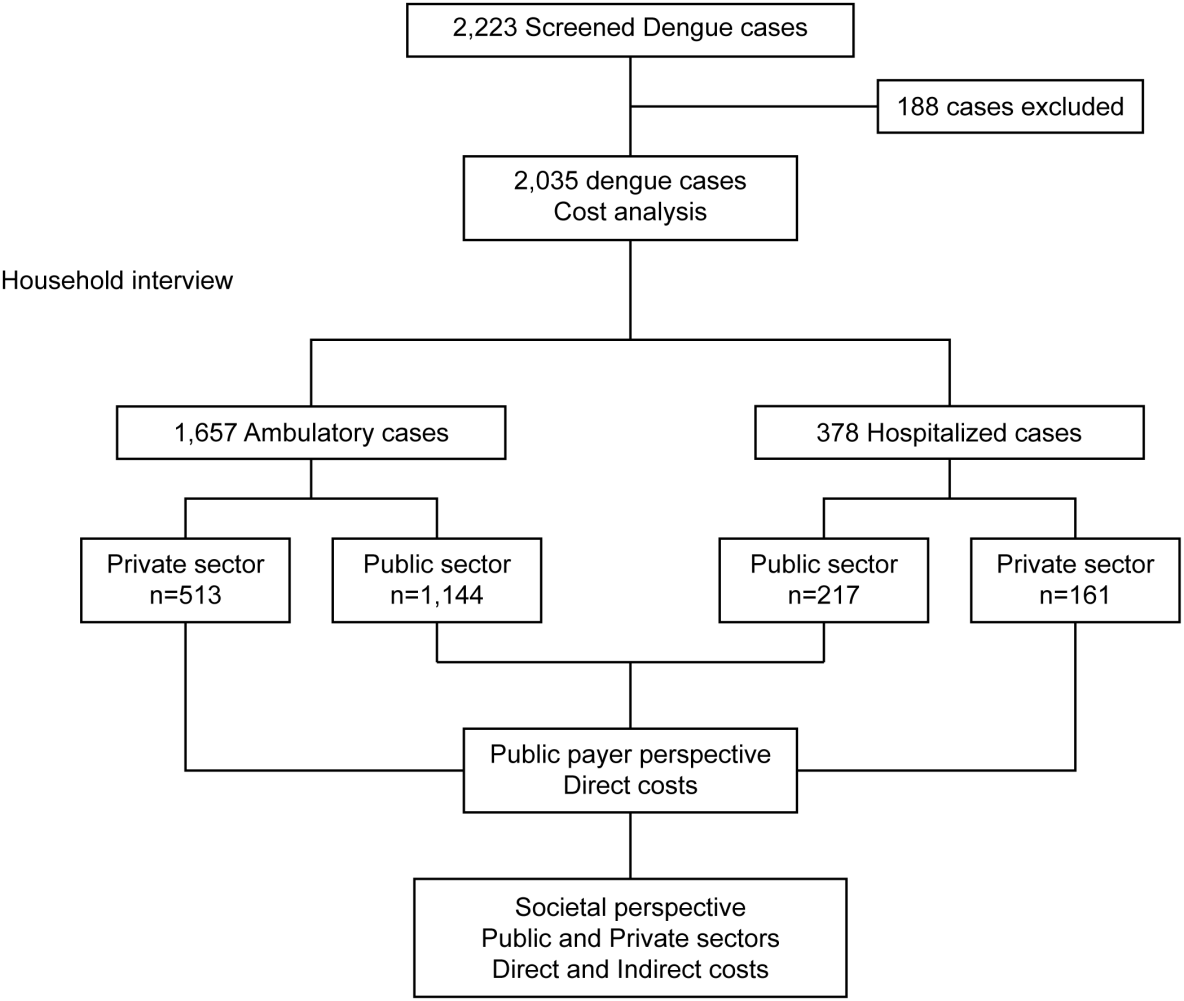
Flowchart of the study design.

Of the 2,223 patients that were screened for dengue, 2,035 (91.5%) symptomatic dengue patients were included in our study (Fig 2). Patients were excluded if they did not provide signed informed consent or if their residential address could not be located/accessed. The number of dengue cases recruited ranged from 279 in the Southeast and North (Rio de Janeiro and Belem) to 415 in the Northeast (Teresina) (Table 2). Approximately 80% of the cases were ambulatory. The difference in the percentage of hospitalized cases between sites was statistically significant (X^2^ = 94.3; df = 5, p<0.001). The residual analysis showed that the proportions of hospitalized patients were similar in the Northeast and North regions (Teresina: 14.5%; Recife: 12.8%; Belem: 18.3%); the highest proportions were found in Goiania (29.5%) and Belo Horizonte (23.3%) (Table 2).

**Table 2:** Characteristics of suspected dengue cases recruited by site in the four regions in Brazil.

Variables	All cities	Midwest	Southeast		Northeast		North
		Goiania	Belo Horizonte	Rio de Janeiro	Teresina	Recife	Belem
**Patient recruited, n**	2,035	387	378	279	415	297	279
**Ambulatory, n (%)**	1,657 (81.4)	273 (70.5)	290 (76.7)	252 (90.3)	355 (85.5)	259 (87.2)	228 (81.7)
**Public sector, n (%)**	1,144 (69.0)	144 (52.7)	123 (42.4)	170 (67.5)	324 (91.3)	155 (59.8)	228 (100.0)
**Hospitalized, n (%)**	378 (18.6)	114 (29.5)	88 (23.3)	27 (9.7)	60 (14.5)	38 (12.8)	51 (18.3)
**Public sector, n (%)**	217 (57.4)	72 (63.2)	71 (80.7)	0	30 (50.0)	27 (71.1)	17 (33.3)
**Age group (years), n (%)**							
0–14	398 (19.6)	64 (16.5)	59 (15.6)	36 (12.9)	69 (16.6)	105 (35.4)	65 (23.3)
≥15	1,637 (80.4)	323 (83.5)	319 (84.4)	243 (87.1)	346 (83.4)	192 (64.6)	214 (76.7)
**Female, n (%)**	1,167 (57.3)	220 (56.8)	224 (59.3)	160 (57.3)	245 (59.0)	162 (54.5)	156 (55.9)
**Education level (≥5 years)** ^**1**^ **, n (%)**							
Illiterate/incomplete elementary	262 (13.7)	44 (11.9)	42 (11.4)	33 (12.0)	51 (13.2)	48 (18.0)	44 (17.2)
Primary school or less	765 (40.0)	148 (39.9)	107 (29.1)	125 (45.5)	164 (42.6)	101 (38.0)	120 (47.0)
Secondary school	626 (32.7)	143 (38.5)	128 (34.8)	78 (28.4)	121 (31.4)	77 (29.0)	79 (30.9)
College or more	261 (13.6)	36 (9.7)	90 (24.5)	33 (12.0)	49 (12.7)	40 (15.0)	13 (4.9)
**Income group** ^**2**^ **, n (%)**							
A1/A2	91 (4.3)	13 (3.4)	41 (9.5)	8 (2.9)	10 (2.4)	16 (5.3)	3 (1.0)
B1	194 (9.3)	41 (10.6)	73 (17.0)	19 (6.8)	15 (3.6)	29 (9.7)	17 (5.9)
B2	406 (19.4)	107 (27.6)	100 (23.3)	52 (18.6)	66 (15.9)	58 (19.5)	23 (8.0)
C1	551 (26.3)	136 (35.1)	110 (25.6)	78 (28.0)	92 (22.2)	77 (25.8)	58 (20.1)
C2	488 (23.3)	66 (17.1)	72 (16.7)	64 (22.9)	139 (33.5)	64 (21.5)	83 (28.8)
D/E	272 (13.0)	24 (6.2)	31 (7.2)	35 (12.5)	92 (22.1)	42 (14.1)	48 (16.7)

^1^ Level of education: Illiterate/Incomplete elementary; Primary school or less: complete elementary 1, complete elementary 2; Secondary school; college or more.

^2^ Economic class by CCEB 2012 (Economic Criterion Classification Brazil)—www.abep.org

Overall, the adult population (≥ 15 years) constituted around 80% of recruited cases. The proportion of dengue cases in the adult population by site ranged from 64.6% in Recife to 87.1% in Rio de Janeiro. There was a significant difference in the proportion of dengue cases in the adult population reported between all sites (X^2^ = 65.4; df = 5, p<0.001). All sites recruited a similar proportion of dengue cases in the age group 0–14 years, except Recife where higher percentage of children and adolescents (35.4%) were recruited (X^2^ = 3.5; df = 5, p = 0.6). The female:male ratio per site was about 3:2(Table 2).

The cost per ambulatory and hospital dengue case across the four regions, from the societal perspective, stratified by public and private sector and by site are summarized in Table 3. Direct costs for ambulatory cases varied from US$ 31 (Rio de Janeiro) to US$89 (Belo Horizonte) in the public sector and constituted the lower share of the total costs for each site except Belo Horizonte. In the private sector, the direct costs varied from US$ 77 (Recife) to US$168 (Goiania). Results from the Anova and Kruskall-Wallis tests (<0.05) showed significant differences between cities in terms of costs per ambulatory dengue case. For ambulatory patients in the public sector two similar subgroups were identified, one group with lower costs (Belem, Teresina and Rio de Janeiro) and the other the higher cost (Belo Horizonte and Goiania), based on the Tamhane post-hoc test. The city of Recife was associated with both subgroups with P value <10%.

**Table 3:** Cost per ambulatory and hospitalized dengue case across four regions in Brazil, societal perspective (2013 US$).

Region City	Public sector			Private sector		
	Direct cost ^1^	Indirect cost	Total cost	Direct cost	Indirect cost	Total cost
	mean (SD)	mean (SD)	mean (SD)	mean (SD)	mean (SD)	mean (SD)
**Ambulatory cases**						
**Midwest**						
Goiania	55 (35)	102 (112)	157 (124)	168 (109)	165 (216)	333 (268)
**Southeast**						
Belo Horizonte	89 (144)	58 (88)	148 (162)	159 (195)	107 (210)	266 (273)
Rio de Janeiro	31 (22)	32 (41)	62 (49)	91 (99)	119 (297)	210 (359)
**Northeast**						
Teresina	33 (45)	39 (92)	72 (109)	133 (99)	367 (522)	500 (550)
Recife	39 (33)	61 (98)	100 (112)	77 (46)	125 (196)	202 (206)
**North**						
Belem	38 (59)	28 (95)	66 (115)	na	na	na
**Hospital cases** ^**2**^						
**Midwest**						
Goiania	227 (16)	171 (234)	398 (236)	450 (205)	119 (141)	569 (233)
**Southeast**						
Belo Horizonte	376 (268)	103 (194)	479 (336)	476 (277)	78 (148)	553 (257)
Rio de Janeiro	na	na	na	906 (388)	56 (191)	962 (451)
**Northeast**						
Teresina	198 (124)	72 (139)	269 (194)	671 (471)	160 (310)	831 (549)
Recife	212 (81)	52 (61)	264 (96)	677 (359)	900 (1,595)	1,577 (1,572)
**North**						
Belem	238 (70)	na	238 (70)	318 (164)	na	318 (164)

na: not available.

^1^ Direct cost includes medical visits, medications and exams, and non-medical costs (food, lodging, and transportation and other out-of-pocket expenses).

^2^ Public sector—Direct cost = Hospital direct cost plus direct cost per hospital case treated at ambulatory weighted by the percent of hospitalized patients consuming ambulatory care: Goiania (97.2%); Belo Horizonte (70.4%); Teresina (60.0%); Recife (55.6%); Belem (70.0%). Private sector—Direct cost = Hospital direct cost plus direct cost per hospital case treated at ambulatory weighted by the percent of hospitalized patients consuming ambulatory care: Goiania (95.2%); Belo Horizonte (23.5%); Rio de Janeiro (50.0%); Teresina (70.0%); Recife (100.0%); Belem (50.0%)

The total cost for hospitalized patients in the public sector varied from US$238(SD 70) in Belem to US$479 (SD 336) in Belo Horizonte. In the private sector, the total cost varied from US$ 318 (SD 164) in Belem to US$ 1,577 (SD 1,572) in Recife. Direct costs constituted the higher share of the total costs for hospitalized cases in both public and private sectors, except for Recife. Two main subgroups of cities were identified according to direct costs for hospitalized dengue patients in the public sector: the lowest direct cost were calculated for three sites (Belem, Teresina and Recife), and highest direct cost for two sites (Belo Horizonte and Goiania), according to the Tamhane *post-hoc* test.

**Fig 3:**
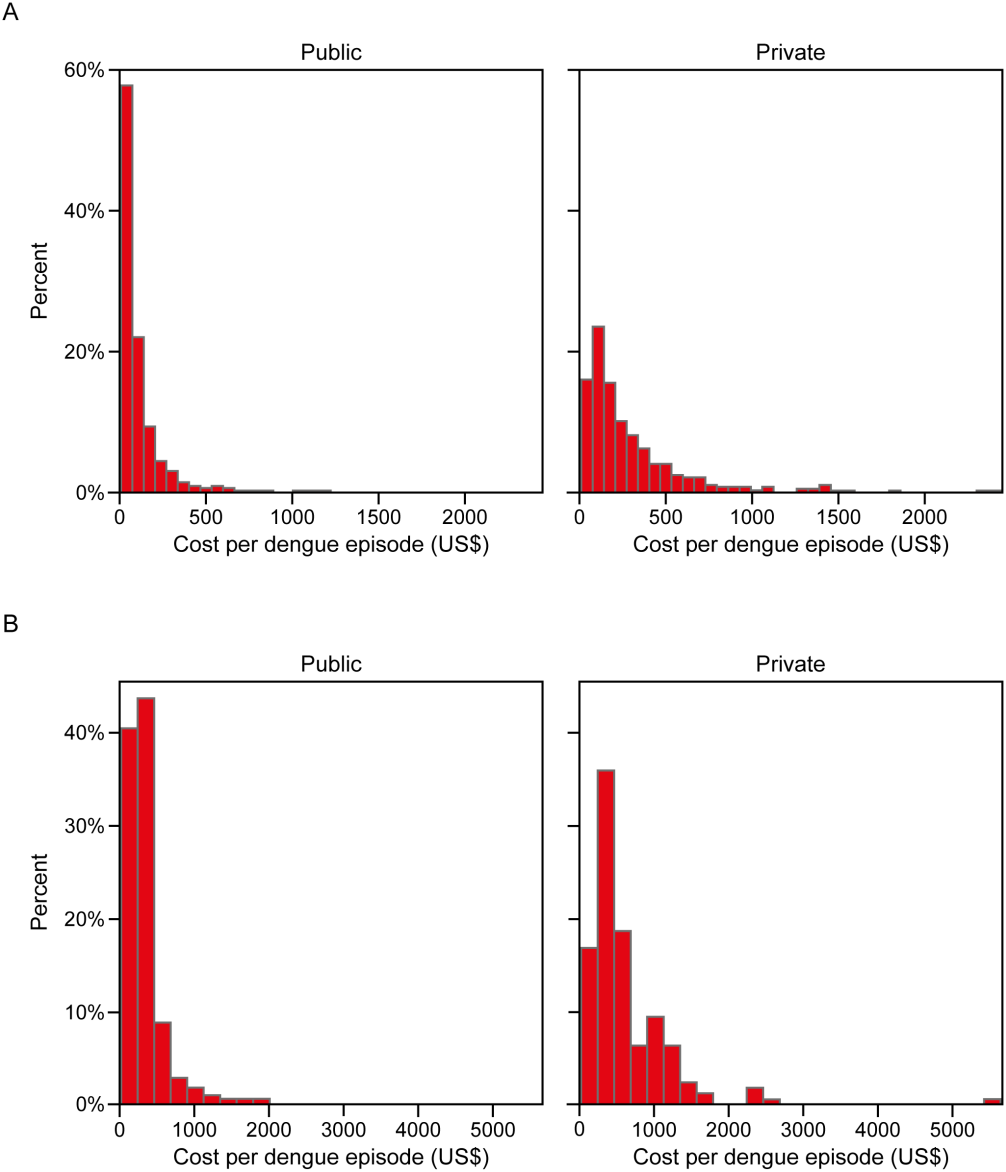
Cost distribution of dengue episode by type of care and health sector (2013 US$).

Fig 3 shows the highly-skewed cost data reported for the ambulatory and hospital cohorts stratified by public or private sector. The majority (approximately 80%) of the ambulatory cases treated in the public sector incurred costs of less than US$ 220 per dengue episode. Costs associated with ambulatory cases treated in the private sector were also skewed: few patients had costs higher than US$ 440. For the hospitalized cohort, more than 80% of the dengue episodes cost US$440 or less in the public sector.

The higher incidence of dengue during the study period was observed in the Southeast and Midwest region of the country, with more than 50 cases for every 1,000 inhabitants (Table 1). The estimated costs of dengue in Brazil presented in Table 4 took into account the uneven distribution of cases between regions as well as differences in dengue management costs. The adjusted direct costs (observed costs weighted by the number of reported cases for each site (see S2 Text) of a dengue episode for ambulatory and hospitalized cases was US$ 64 (90% CL: 48–80) and US$ 237 (90% CL: 177–297), respectively. Extrapolating our estimates to national surveillance system data, we estimated the annual total cost for dengue at US$ 164 million (90% CL: 123–205) from the public payer perspective. This figure increases to an estimated US$ 447 million (90% CL: 335–559) when we adjusted for underreporting (EF = 3.2 for ambulatory cases and EF = 1.6 for hospitalized cases). From the societal perspective, we estimated the annual total cost for dengue at US$ 404 million (90% CL: 301–508) or US$ 1,147 million (90% CL: 885–1,445), the latter figure also estimated with EFs for possible under-reporting (Table 4). These annual total estimated cost for dengue correspond to per capita costs of US$ 6.7or US$ 19.0 (with EFs) from the societal perspective based on the labor force population (60.2 million, IBGE 2011). In Brazil, there were an average 529 deaths due to dengue and an average of 944,733 notified dengue cases (SINAN, 2011–2013), with a fatality rate of 0.056% (90% CL: 0.051–0.061). At the national level, the total cost for fatal a dengue episode was approximately US$ 65 million (90% CL: 48–81); US$ 34 million for males and US$ 31 million for females. We estimated the cost of a fatal dengue case as US$ 122,477.41 (Table 4).

**Table 4:** Estimated costs of dengue in Brazil from the public payer and societal perspectives, considering two scenarios (2013 US$).

	Scenario 1	Scenario 2
**Reported Dengue Cases Brazil (SINAN)**		
Ambulatory	1,8 million cases	5,8 million cases (EF = 3.2)
Hospital	201 thousand cases	322 thousand cases (EF = 1.6)
**Public payer perspective**		
Ambulatory		
Estimated direct cost per case—US$ (90% CL)	$ 64 (48–80)	
Cost—US$ (90% CL)	$ 116 million (87–145)	$ 371 million (278–464)
Hospital		
Estimated direct cost per case—US$ (90% CL)	$ 237 (177–297)	
Cost—US$ (90% CL)	$ 48 million (36–60)	$76 million (57–96)
**Total cost—US$** (90% CL)	**$ 164 million (123–205)**	**$ 447 million (335–559)**
**Societal perspective**		
Ambulatory		
Estimated cost per case—US$ (90% CL)	$ 173 (129–218)	
Cost—US$ (90% CL)	$ 314 million (234–395)	$ 1,003 million (748–1,264)
Hospital		
Estimated cost per case—US$ (90% CL)	$ 448 (333–562)	
Cost—US$ (90% CL)	$ 90 million (67–113)	$ 144 million (107–181)
**Total cost—US$** (90% CL)	**$ 404 million (301–508)**	**$ 1,147 million (885–1,445)**
Number of fatal cases	529 (395–665)	
Fatal costs	65 million (48–81)	
**Grand total cost**	**468 million (349–590)**	**1,212 million (904–1,526)**

Scenario 1: official national reported dengue cases, SINAN (September 2012 –August 2013)

Scenario 2: official national reported dengue cases, SINAN (September 2012 –August 2013) with expansion factor (EF ambulatory = 3.2; EF hospital = 1.6)

CL: Certainty Level

**Fig 4:**
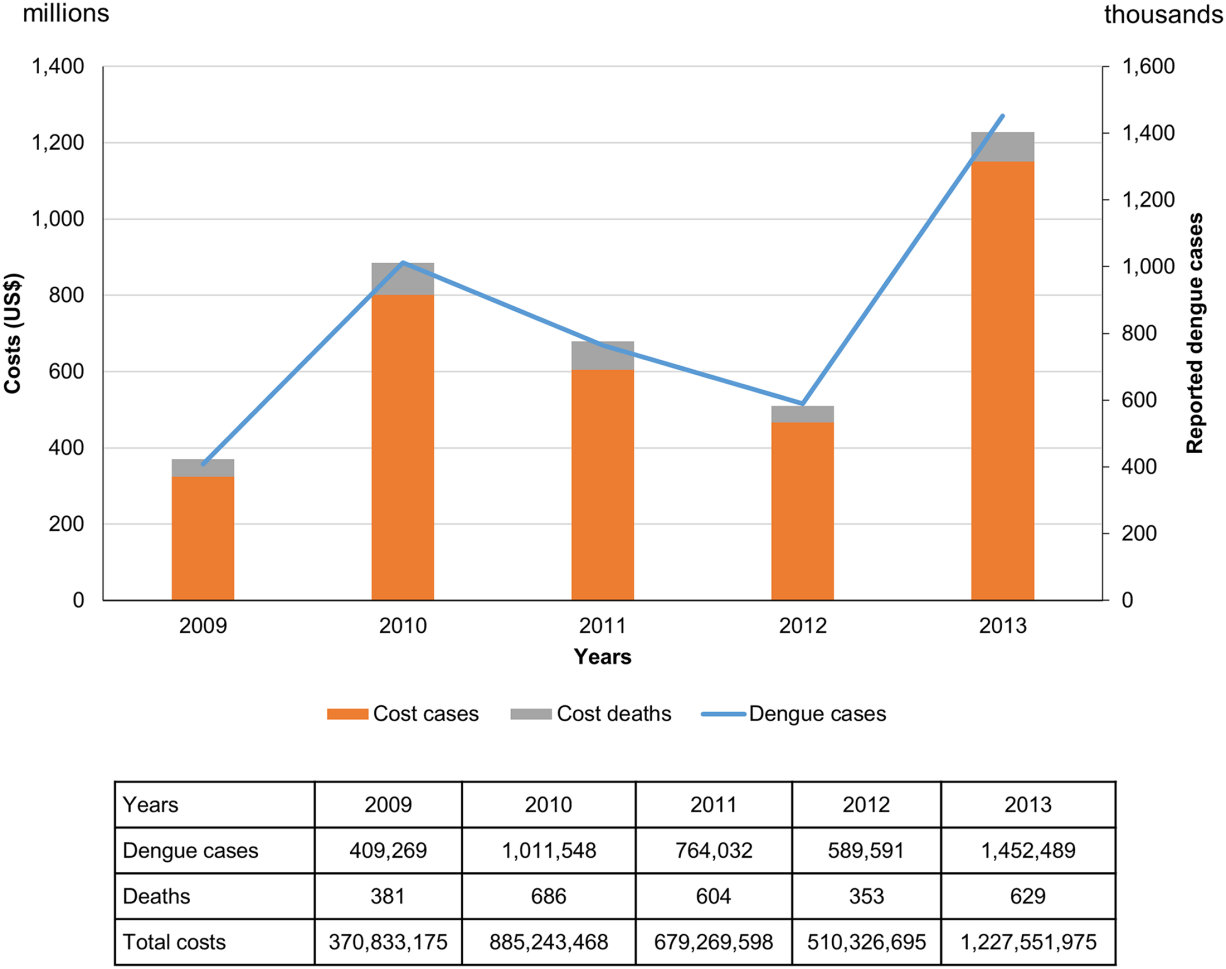
Estimated cost of dengue by reported number of cases in the period 2009–2013 (2013 US$). The cost of dengue was estimated considering the ratio of ambulatory:hospital (9:1). These costs do not include the cost of vector control neither the cost of the surveillance system. Estimated number of reported dengue cases were based on online surveillance data (SINAN, 2009–2013), with EFs.

Fig 4 shows the reported dengue cases and the burden of dengue from 2009 to 2013. In this five years period, the reported dengue cases was the lowest (409 thousand cases) in the year 2009, increased to more than 1 million cases in the epidemic year of 2010 and reached 1.4 million in the year 2013. Therefore, the total costs depends upon the number of reported dengue cases and deaths, varied from US$ 371 million (2009) up to US$ 1,228 million (2013).

## Discussion

Our study is the first multi-center study to assess the cost of dengue illness across four dengue-endemic Brazilian regions, taking into account ambulatory and hospitalized cases in both the public and private sectors. The total economic burden of dengue in Brazil from a societal perspective was estimated at US$ 468 million (90% CL: 349–590) (with no adjustment for underreporting), of which, the majority (67%) was associated with ambulatory cases. Indirect costs (i.e. lost productivity) constituted the higher share of the total costs for ambulatory cases in both public and private sectors, consistent with a previous cost evaluation of the economic burden of dengue episodes in Brazil [10]. Moreover, higher total costs for ambulatory cases were observed in wealthier regions in our study which might have been due to, for example, more laboratory tests and lost productivity. From the public payer perspective, the total economic burden was estimated at US$ 164 million (90% CL: 123–205 million) (with no adjustment for underreporting).

We adopted a similar protocol to a previous cost evaluation of the economic burden of dengue episodes in Brazil, which included the city of Goiania, as part of an international cost of dengue study that included five countries in the Americas [9, 10]. However, the previous study applied unit cost for health services using only private sector values [9]. Therefore, the costs per case for ambulatory and hospitalized cases were higher in the earlier study compared to our results. Our data clearly show higher costs in the private sector compared to the public sector for both ambulatory and hospitalized cases. However, the estimates for the public sector ambulatory visit may be conservative even when applying the highest value of the medical visit reimbursed by the Unified Health System (SUS).

Our study is at least, in part, consistent with a recent retrospective cross-sectional census study by Vieira Machado et al (2014) on hospitalized dengue patients in the public and private Brazilian health sectors in Dourados City, Mato Grosso do Sul State [33]. The latter study reported direct mean medical costs per hospitalized dengue case in the public sector of US$ 510 (SD, 1,135) (values adjusted for inflation for the year 2013), compared with US$ 198–376 in our study. Vieira Machado et al also reported higher mean direct medical cost of US$ 1,193 (SD, 2,701) (values adjusted for inflation for the year 2013) per hospitalized dengue case in the private sector relative to the public sector, compared with US$ 318–906 in our study. Of note, their study was based on a sample of 288 laboratory-confirmed dengue cases, whereas our study was based on a much broader case definition that included 2,035 suspected dengue cases. In addition, we recruited cases from different geographic areas in Brazil from the study conducted by Vieira Machado et al.

The current economic evaluation was conducted concurrently with major dengue outbreaks in the Southeast and Midwest regions (Belo Horizonte and Goiania), in the period August 2012 September 2013. Approximately 100,000 probable dengue cases were registered in the city of Belo Horizonte in the Southeast region and about 56,000 cases in Goiania in the Midwest region. However, there was a decrease in the incidence of dengue cases and hospitalizations reported in the city of Rio de Janeiro compared to previous years. In addition, the cities of Belem (North), Recife and Teresina (Northeast) had a reduction in both incidence and hospitalizations for dengue. As such, we evaluated the economic burden in areas that included both dengue outbreaks and low virus circulation. This approach, including settings with high and low virus transmission, is recommended by the dengue guidelines for cost evaluation [12].

Our study has both strengths and weaknesses that need to be considered when making generalizations for the whole of Brazil. A strength of our economic burden study is that we included several types of health facilities in both private and public sectors across four dengue-endemic regions in Brazil in order to provide more representative cost estimates of the population assessed. Moreover, the cases recruited to our study had a similar gender and age distribution across the regions, and were in line with data obtained in the official notification system for Brazil, suggesting that they were representative of the population in those regions. They also had similar income level distributions to the general population in their region according to census data. Another strength was that we designed our study in line with current guideline recommendations [12].

The main limitation of our study is that the total cost depends largely on number of dengue cases registered in the passive surveillance system in Brazil (SINAN). Other drawbacks of using passive surveillance systems in economic burden studies include: selection bias; data may not be well suited for economic study; data gathered for other purposes; and inherent underlying confounding factors. Nonetheless, passive surveillance systems are a readily available source of data that is representative of the national sample of suspected dengue cases and as such, maximizes external validity. It is also well known, that the number of symptomatic dengue cases and its geographical location, like most vector-borne disease, varies each year. As such, the ambulatory:hospitalized ratio would vary according to the burden of the disease during dengue outbreaks or periods of low virus transmission rates. The ambulatory:hospitalized case ratio could also be affected by the implementation of temporary health structures (tents or stabilization wards) during outbreaks, as observed in Belo Horizonte in 2013 [34].

In addition, our study focused on the economic burden of dengue in urbanized areas of Brazil, and as such, may not be extrapolated to less-urbanized areas. The cost estimates obtained in our study pertain to a one year study period and outbreaks were reported in some specific capital cities. There was no concerted effort to estimate the costs associated with dengue outbreaks in this study. To fully cost dengue outbreaks, we would have needed to account for the various stages of the outbreaks—before, during and after an outbreak. For indirect costs we estimated only the loss of income of patients who perceived had a monetary income in the previous month. For example, if the dengue patient was a housekeeper and did not perceive a monetary income, their illness would still present an economic loss to the economy but would likely not have been captured in our analysis and thus would have led to an underestimation of indirect costs. Finally, it was beyond the scope of the present study to include cost components related to preventive strategies such as vector control, routine activities, recurrent costs, and operational costs, as well as the cost components related to outbreak control, community mobilization and tourism.

To place the cost estimates of the epidemic year (in the current study) in temporal perspective, we also estimated the global costs of dengue for cases registered in the five years up to 2013. The historical series of dengue cases reported provides an example of the large annual variation in the number of registered cases, and whether they can be considered epidemic or endemic years. Our results underscore the need for regular national cost evaluation, preferably alongside the surveillance system. However, the under-reporting or the over-reporting of dengue cases by the surveillance system are constant issues raised in the literature and by policy makers. We opted to estimate the cost of dengue for suspected cases instead of the laboratory-confirmed cases in accordance with current guidelines for cost estimates of costs [12].

In Brazil, there has been a decrease in age-standardized incidence and deaths from malaria in the last decade. Around 130,000 malaria cases and 71 associated deaths were registered in the 2013 [35]. In contrast, there has been a striking increase in dengue in the last decade with over 1 million cases and over 500 deaths registered in the 2013. Of note, dengue transmission has now reached most of the regions in the country but malaria is currently restricted to the Amazon basin region [35]. The comparison of the cost of dengue with other infectious disease in Brazil is hampered since there are few cost studies of epidemic diseases nationwide.

In summary, our study shows that the economic burden associated with dengue in Brazil is substantial. We hope our study will help policy makers to inform their decisions when setting goals and priorities, and assessing estimates of the impact of any proposed interventions in the management of dengue. We believe that our results are a timely estimate of the cost of a dengue episode in Brazil, considering both the public payers and the societal perspective.

## Supporting Information

S1 Table Data sources used to calculate direct and indirect costs for ambulatory dengue cases. (DOCX)

S2 Table Data sources used to calculate direct and indirect costs for hospitalized dengue cases. (DOCX)

S1 Text Questionnaires. (DOCX)

S2 Text Assumptions to estimate cost per dengue case using primary data from six cities and extrapolating to national level. (DOCX)
