# Supplementary material for: Economic Impact of Dengue: Multicenter Study across Four Brazilian Regions
Source: PLoS Negl Trop Dis. 2015 Sep 24;9(9):e0004042. doi: 10.1371/journal.pntd.0004042 (PMC4581827; doi:10.1371/journal.pntd.0004042)
Supplement: S1 Table — (DOCX) [file pntd.0004042.s001.docx]

**S1 Table.** Data sources used to calculate direct and indirect costs for ambulatory dengue cases

| **Direct costs (Cost_Direct_ambulatory_)** | | | |
| --- | --- | --- | --- |
|  | **Data source** | **Public** | **Private** |
| Medical visits (Cost_Medicalvisit) | Household interview: Chronological order of medical visits during the dengue episode; Public sector: SUS value for one medical visit in emergency; Private sector: health insurance plan for one medical visit (Unimed) | Nº medical visits *X* value paid by SUS | Nº medical visits *X* value paid by the health insurance plan |
| Medication (Cost_Medication) | Household interview: self-report of regimen of drugs taken/prescribed during dengue episode; Public sector: medication costs for public purchase (Table CMED, <http://portal.anvisa.gov.br>); Private sector: medication list (Guia Farmaceutico Brasindice, January, 2013) | Medication used *X* quantity *X* price | Medication used *X* quantity *X* price |
| Laboratory tests/exams (Cost_Labexams) | Household interview: self-report type of laboratory test and/or exams performed during dengue episode; Public sector: DataSUS online table (<http://sigtap.datasus.gov.br>); Private sector: Classificação Brasileira Hierarquizada - procedimentos Medicos – Table AMB- Associacao Medica Brasileira (<http://www>.amb.org.br/_arquivos/_downloads/ cbhpm_2012.pdf) | Type of laboratory test *X*  quantity *X* price | Type of laboratory test *X*  quantity *X* price |
| Direct non-medical costs (Cost_NMDirect) | Household interview: self-report of monetary value expended in each health activity during dengue episode | ∑ food, lodging, and transportation and other out-of-pocket expenses | ∑ food, lodging, and transportation and other out-of-pocket expenses |
| **Indirect costs (Cost_Indirect_ambulatory_)** | | | |
|  | **Data source** | **Public** | **Private** |
| Loss of income by patient by patient or care-giver | Household interview: Reported number of days of work lost by patient and/or caregivers and the monetary value of the previous month’s income | Nº days lost *X* value of day work | Nº days lost *X* value of day work |
| School time lost by patient or care-giver | Household interview: History of days of schooling lost by patient and/or caregivers during dengue episode; ^a^Diario Oficial da Uniao - Portaria Interministerial Nº 1360-A, 19 de novembro de 2012 (accessed at: <http://www.jusbrasil.com.br/diarios/DOU/2012/11/19>); College education: based on<http://ultimosegundo.ig.com.br/educacao/educacao+basica+custa+mais+na+particular+superior+na+publica/n1597000724462.html> (annual cost per student R$ 15,000.00/ USD 6.612.00) | Nº days lost *X* estimated value according to the education level and region | Nº days lost *X* estimated value according to the education level and region |

^a^Brazilian criterion establishes the cost of school that takes into account the level of education in each State.
