# Supplementary material for: Economic Impact of Dengue: Multicenter Study across Four Brazilian Regions
Source: PLoS Negl Trop Dis. 2015 Sep 24;9(9):e0004042. doi: 10.1371/journal.pntd.0004042 (PMC4581827; doi:10.1371/journal.pntd.0004042)
Supplement: S1 Text — (DOCX) [file pntd.0004042.s003.docx]

**Economic Impact of Dengue: Multicenter Study Across Four Brazilian Regions**

Martelli et al, 2015, PLOS Neglected Tropical Diseases

**S1 Text.** Questionnaires

We collected individual data using the following questionnaires:

1. Phase 1. “Formulario A” that corresponds to screening interview with personal data.
2. Phase 2. “Formulario B” used for the household interview.
3. For hospitalized patients, we also extracted data for the patients’ medical record using the “Formulario D”.

| \|  \| \| --- \|   Número do Paciente  **Impacto Econômico e na Qualidade de Vida associados à Dengue no Brasil**  **2012/2013** |
| --- | --- |

**FORMULÁRIO A**

**10000 - Triagem inicial**

| **Q1001** | O paciente / Responsável concordou/ assinou TCLE? | | | 1. Sim | | 2. Não | |
| --- | --- | --- | --- | --- | --- | --- | --- |
| **Q1003** | Número do paciente: | | |  | | | |
| **Q1016** | Data de início dos sintomas: | | | */ /* | | | |
| **Q1004** | Marcar data de entrevista domiciliar ~15 a 20 dias após do início dos sintomas: | | | */ /* | | | |
| **Q1005** | CPF do entrevistador: | | |  | | | |
| **Q1006** | Data da triagem | | | */ /* | | | |
| **Q1007** | Município | | |  | | | |
| **Q1008** | Unidade de saúde | | |  | | | |
| **Q1009** | Número de registro do paciente na Unidade de Saúde: | | |  | | | |
| **Q1010** | Tipo de Unidade de saúde: | | | 1. Ambulatório | | 2. Hospital | |
| **Q1011** | Setor da Unidade de Saúde: | | | 1. Público | | 2. Privado | |
| **Q1012** | Nome do paciente: | | |  | | | |
| **Q1013** | Data de nascimento: | | | */ /* | | | |
| **Q1014** | Sexo: | | | 1. Feminino | | 2. Masculino | |
| **Q1015** | Apresenta/ apresentou sintomas de dengue? | | | 1. Sim | | 2. Não | |
| **Q1017** | Você teve/tem febre? | | | 1. Sim | | 2. Não | |
| **Q1018** | Nome do responsável (se menor): | | |  | | | |
| **Q1019** | Endereço 1: | | |  | | | |
| **Q1020** | Ponto de referência 1: | | |  | | | |
| **Q1021** | Endereço 2: | | |  | | | |
| **Q1022** | Ponto de referência 2: | | |  | | | |
| **Q1023** | Telefone 1: | | *Telefone 2:* | | *Telefone 3:* | | Telefone 4: |
| **Q1024** | Observações: |  | | | | | |

**FORMULÁRIO B**

**2000. Características demográficas do paciente**

| **Q2001** | Número do paciente: |  |
| --- | --- | --- |
| **Q2002** | Data da entrevista domiciliar: | */ /* |

**Se está entrevistando o paciente, marque a opção 9 e vá para a Q.2004**

| **Q2003** | Se você está entrevistando um responsável, qual é a relação do responsável com o paciente? | 1. Mãe | 1. Pai | | 1. Avó | 1. Avô | | 1. Irmão maior | | 1. Outro | 9. Não se aplica | |
| --- | --- | --- | --- | --- | --- | --- | --- | --- | --- | --- | --- | --- |
| **Q2004** | Qual é o nível de escolaridade do paciente? | 1. Analfabeto/ Fundamental 1 incompleto | | 1. Fundamental 1 completo/ Fundamental 2 incompleto | | | 1. Fundamental 2 completo/ Médio incompleto | | 1. Médio completo/ Técnico/ Superior incompleto | | | 1. Superior completo |
| **Q2005** | Qual é atualmente o tipo de emprego do paciente? | 1. Funcionário público | | 1. Empregado não governamental | | | 1. Autônomo | | 1. Empresário/Patrão | | | 1. Não tem emprego remunerado |
| **Q2007** | Qual o estado civil do paciente? | 1. Solteiro | | 1. Casado | | | 1. Divorciado | | 1. Viúvo | | | 1. União estável |

**INFORMAÇÃO DO RESPONSÁVEL, preencher para pacientes menores de idade.**

| **Q2008** | Qual a idade do responsável (em anos)? |  | | | | | |
| --- | --- | --- | --- | --- | --- | --- | --- |
| **Q2009** | Qual é o nível de escolaridade do responsável? | 1. Analfabeto/ Fundamental 1 incompleto | 1. Fundamental 1 completo/ Fundamental 2 incompleto | 1. Fundamental 2 completo/ Médio incompleto | 1. Médio completo/ Técnico/ Superior incompleto | | 1. Superior completo |
| **Q2010** | Qual é atualmente o tipo de emprego do responsável? | 1. Funcionário público | 1. Empregado não governamental | 1. Autônomo | 1. Empresário/Patrão | 1. Não tem emprego remunerado | |

**Posse de itens (Critério de Classificação Econômica Brasil - CCEB)**

| **Q2012** | A residência do paciente tem televisão em cores? | 0 | 1 | 2 | 3 | 4 ou + |
| --- | --- | --- | --- | --- | --- | --- |
| **Q2013** | A residência do paciente tem rádio? | 0 | 1 | 2 | 3 | 4 ou + |
| **Q2014** | A residência do paciente tem banheiro? | 0 | 1 | 2 | 3 | 4 ou + |
| **Q2015** | A residência do paciente tem automóvel? | 0 | 1 | 2 | 3 | 4 ou + |
| **Q2016** | A residência do paciente tem empregada mensalista? | 0 | 1 | 2 | 3 | 4 ou + |
| **Q2017** | A residência do paciente tem máquina de lavar? | 0 | 1 | 2 | 3 | 4 ou + |
| **Q2018** | A residência do paciente tem videocassete e/ou DVD? | 0 | 1 | 2 | 3 | 4 ou + |
| **Q2019** | A residência do paciente tem uma geladeira? | 0 | 1 | 2 | 3 | 4 ou + |
| **Q2020** | A residência do paciente tem freezer (aparelho independente ou parte da geladeira duplex)? | 0 | 1 | 2 | 3 | 4 ou + |
| **Q2021** | A residência do paciente tem aspirador de pó? |  |  |  |  |  |

**3000. MANIFESTAÇÕES CLÍNICAS, MEDICAMENTOS E LABORATÓRIO.**

| **Q3001** | Quando foi o início dos sintomas? | */ /* | |
| --- | --- | --- | --- |
| **Q3002** | Você está com algum sintoma no momento da entrevista? | 1. Sim | 1. Não |
| **Q3000-X2** | Data em que você se recuperou completamente. (apenas se não tiver mais sintomas da Dengue) | */ /* | |
| **Q3003** | Você teve febre no período da doença? | 1. Sim | 1. Não |

**Você teve algum dos sintomas ou sinais durante o período da doença?**

| **Q3004** | Dor de cabeça | 1. Sim | 1. Não | 1. Não sabe |
| --- | --- | --- | --- | --- |
| **Q3005** | Dor nos olhos | 1. Sim | 1. Não | 1. Não sabe |
| **Q3006** | Náuseas | 1. Sim | 1. Não | 1. Não sabe |
| **Q3007** | Vômitos | 1. Sim | 1. Não | 1. Não sabe |
| **Q3008** | Cansaço/prostração | 1. Sim | 1. Não | 1. Não sabe |
| **Q3009** | Perda de apetite | 1. Sim | 1. Não | 1. Não sabe |
| **Q3010** | Dor muscular ou articular (dor nas juntas) | 1. Sim | 1. Não | 1. Não sabe |
| **Q3011** | Dor nos ossos | 1. Sim | 1. Não | 1. Não sabe |
| **Q3012** | Dor abdominal | 1. Sim | 1. Não | 1. Não sabe |
| **Q3013** | Tontura | 1. Sim | 1. Não | 1. Não sabe |
| **Q3014** | Manchas vermelhas na pele | 1. Sim | 1. Não | 1. Não sabe |
| **Q3015** | Sangramentos (pequenos pontos vermelhos na pele, machucados espontâneos ou provocados, sangramentos pelo nariz, sangramento nas gengivas ou outro tipo de sangramento). | 1. Sim | 1. Não | 1. Não sabe |
| **Q3016** | Coceira | 1. Sim | 1. Não | 1. Não sabe |
| **Q3017** | Diarreia | 1. Sim | 1. Não | 1. Não sabe |
| **Q3018** | Inchaço nos olhos | 1. Sim | 1. Não | 1. Não sabe |
| **Q3019** | Sede excessiva | 1. Sim | 1. Não | 1. Não sabe |
| **Q3020** | Diminuição da urina | 1. Sim | 1. Não | 1. Não sabe |
| **Q3021** | Agitação | 1. Sim | 1. Não | 1. Não sabe |
| **Q3022** | Sonolência/letargia | 1. Sim | 1. Não | 1. Não sabe |
| **Q3023** | Dor de garganta ou secreção nasal | 1. Sim | 1. Não | 1. Não sabe |

**Medicamentos**

| **Q3024** | Durante este período da doença, você recebeu alguma medicação prescrita pelo serviço de saúde ou médico? | 1. Sim | | | 1. Não | | | | | | | | | 1. Não sabe | | |
| --- | --- | --- | --- | --- | --- | --- | --- | --- | --- | --- | --- | --- | --- | --- | --- | --- |
| **Q3025** | Qual(is) medicamento(s) você recebeu? | 1. Med.A: | | 1. Med.B: | | | | 1. Med.C: | | 1. Med.D | | | 1. Med.E | | | 1. Não sabe |
| **Q3026** | Quantos comprimidos/dosagem você tomou durante o período da doença? | 1. Med.A: | | 1. Med.B: | | | | 1. Med.C: | | 1. Med.D | | | 1. Med.E | | | 1. Não sabe |
| **Q3027** | Durante este período da doença, você comprou alguma medicação? | | 1. Sim | | | | 1. Não | | | | | | | 1. Não sabe | | |
| **Q3028** | Qual(is) medicamento(s) você comprou? | | 1. Med. A: | | | 1. Med.B: | | | | | 1. Med.C: | | | | 1. Não sabe | |
| **Q3029** | Quantos comprimidos / dosagem você tomou durante o período da doença? | | 1. Med. A: | | | 1. Med.B: | | | | | 1. Med.C: | | | | 1. Não sabe | |
| **Q3030** | Quanto gastou na compra de medicamentos? | | R$ | | | | | | | | | | | | | |
| **Q3000-X1** | Quanto tempo, depois de notar os primeiros sinais de enfermidade, você recebeu a primeira assistência por parte de um serviço de saúde ou médico? | | 1. No mesmo dia (em 24 horas) | | | | | | 1. No segundo dia (entre 24 e 48 horas) | | | 1. Depois de 48 horas | | | | |

**MANIFESTAÇÕES CLÍNICAS, MEDICAMENTOS E LABORATÓRIO.**

| **Q3031** | Já fez sorologia? | 1. Sim | 1. Não | 1. Não sabe |
| --- | --- | --- | --- | --- |

Se "não/não sabe", vá para a pergunta Q3036.

| **Q3032** | Quantos exames sorológicos você fez? | |  | | | | | | |
| --- | --- | --- | --- | --- | --- | --- | --- | --- | --- |
| **Q3033** | Quando foi coletada a primeira amostra de sangue para sorologia? | |  | | | | | | |
| **Q3034** | Quando foi coletada a segunda amostra de sangue para sorologia? | |  | | | | | | |
| **Q3035** | Qual o resultado da sorologia? | | | | | | | | |
|  | Exame 1, Data: */ /* | Qual o tipo de Exame? | | 1. Reagente | 1. Não reagente | | 1. Inconclusivo | | 1. Não sabe |
|  | Exame 2, Data: */ /* | Qual o tipo de Exame? | | 1. Reagente | 1. Não reagente | | 1. Inconclusivo | | 1. Não sabe |
|  | Exame 3, Data: */ /* | Qual o tipo de Exame? | | 1. Reagente | 1. Não reagente | | 1. Inconclusivo | | 1. Não sabe |
|  | Exame 4, Data: */ /* | Qual o tipo de Exame? | | 1. Reagente | 1. Não reagente | | 1. Inconclusivo | | 1. Não sabe |
|  | Exame 5, Data: */ /* | Qual o tipo de Exame? | | 1. Reagente | 1. Não reagente | | 1. Inconclusivo | | 1. Não sabe |
| **Q3036** | Quantos exames de hemograma realizou? | |  | | | | | | |
| **Q3037** | Fez alguma radiografia? | | 1. Sim | | | 1. Não | | 1. Não sabe | |

Se "não/não sabe", vá para a pergunta Q3040.

| **Q3038** | De qual parte do corpo? (especificar todas as partes do corpo radiografadas) |  | | |
| --- | --- | --- | --- | --- |
| **Q3039** | Quantos exames de RX realizou? |  | | |
| **Q3040** | Fez alguma ultrassonografia? | 1. Sim | 1. Não | 1. Não sabe |

Se "não/não sabe", vá para a pergunta Q3043.

| **Q3041** | De qual parte do corpo? (especificar todas as partes do corpo) |  | | |
| --- | --- | --- | --- | --- |
| **Q3042** | Quantos exames de ultrassonografia realizou? |  | | |
| **Q3043** | Fez algum outro exame? (sangue, urina, tomografia, etc.) | 1. Sim | 1. Não | 1. Não sabe |

Se "não/não sabe", vá para a seção 4000.

| **Q3044** | Qual exame? |  |
| --- | --- | --- |
| **Q3045** | Quantas vezes? |  |
| **Q3046** | Qual outro exame? |  |
| **Q3047** | Quantas vezes? |  |

**A ser preenchido pelo supervisor de campo.**

**Notificação de dengue (por favor, verifique o registro médico do hospital e a ficha de investigação de dengue).**

| **Q3048** | Este caso foi notificado/registrado como um episódio de Dengue? | 1. Sim | 1. Não |
| --- | --- | --- | --- |

Se "não", vá para a pergunta Q3053.

| **Q3049** | Este caso foi confirmado como? | 1. Critério clínico-epidemiológico | | | | | 1. Laboratorialmente | | | | | |
| --- | --- | --- | --- | --- | --- | --- | --- | --- | --- | --- | --- | --- |
| **Q3050** | Com que diagnóstico ele foi notificado/registrado? | 1. Dengue sem classificação | 1. Dengue clássica | | | 1. Dengue clássica com manifestações hemorrágicas | | | 1. Febre hemorrágica do dengue | | | 1. Síndrome do choque do dengue |
| **Q3051** | Pelos exames/sintomas, qual a classificação do caso confirmado, segundo os critérios da OPAS? | 1. Dengue sem sinais de alerta | | 1. Dengue com sinais de alerta | | | | | | 1. Dengue grave | | |
| **Q3052** | Foi isolado o vírus da dengue? | 1. Sim | | | | 1. Não | | | | | 1. Não sabe | |
| **Q3053** | Qual o tipo de vírus isolado? | 1. DV1 | 1. DV2 | | 1. DV3 | | | 1. DV4 | | 1. Não sabe informar | | |

**4000. ASSISTÊNCIA**

**Por favor, especifique a assistência recebida durante o período da doença, em ordem de recebimento desde o início dos sintomas.**

| **Códigos para o tipo de assistência (TA):** | **Códigos para tipos de serviço (TS):** | **Códigos para tipos de transporte:** |
| --- | --- | --- |
| 1. Farmácia 2. Centro/posto/PSF/unidade de saúde   2a. Consulta  2b. Sala de hidratação  2c. Sala de estabilização   1. Consultório 2. Hospital   4a. Sala de urgência/emergência em hospital  4b. Hospitalização em enfermaria  4c. Hospitalização e UTI (cuidados intensivos)   1. Laboratório 2. Banco de sangue 3. Outro | 1. SUS (sistema público) 2. Convênio (sistema suplementar) 3. Particular (pagamento direto pelo paciente) 4. Coparticipação 5. Outro | 1. A pé 2. Bicicleta 3. Moto 4. Carro 5. Ônibus/ micro-ônibus 6. Bicicleta-táxi 7. Moto-táxi 8. Táxi 9. Carona 10. Outro tipo |

| **Q4010** | Você tem/ utilizou seguro de saúde durante esse episódio de dengue? | 1. Sim | 1. Não | 1. Não sabe |
| --- | --- | --- | --- | --- |

Se "não", vá para a seção 5000.

| **Q4011** | Se sim, qual o plano ou seguro de saúde? |  |
| --- | --- | --- |

|  | **Ordem de assistência recebida** | **Assistência** | | | | | | **Transporte (sentido único) à Assistência** | | | | **Alimentação** | **Alojamento** |
| --- | --- | --- | --- | --- | --- | --- | --- | --- | --- | --- | --- | --- | --- |
|  |  | Tipo de assistência (TA)  (a) | Tipo de serviço (TS) (b) | Tempo de espera (min) (c) | Tempo de consulta (min) (d) | Valor total gasto (e) | Valor reembolsado ao paciente (f) | Tipo de transporte (g) | Distância (Km) (h) | Tempo de viagem (min) (i) | Valor total do gasto (j) | Valor total do gasto (k) | Valor total do gasto (k) |
| **Q4001** | 1º |  |  |  |  |  |  |  |  |  |  |  |  |
| **Q4002** | 2º |  |  |  |  |  |  |  |  |  |  |  |  |
| **Q4003** | 3º |  |  |  |  |  |  |  |  |  |  |  |  |
| **Q4004** | 4º |  |  |  |  |  |  |  |  |  |  |  |  |
| **Q4005** | 5º |  |  |  |  |  |  |  |  |  |  |  |  |
| **Q4006** | 6º |  |  |  |  |  |  |  |  |  |  |  |  |
| **Q4007** | 7º |  |  |  |  |  |  |  |  |  |  |  |  |
| **Q4008** | 8º |  |  |  |  |  |  |  |  |  |  |  |  |
| **Q4009** | 9º |  |  |  |  |  |  |  |  |  |  |  |  |
| **Q4010x** | 10º |  |  |  |  |  |  |  |  |  |  |  |  |
| **Q4011x** | 11º |  |  |  |  |  |  |  |  |  |  |  |  |
| **Q4012x** | 12º |  |  |  |  |  |  |  |  |  |  |  |  |
| **Q4013x** | 13º |  |  |  |  |  |  |  |  |  |  |  |  |
| **Q4014x** | 14º |  |  |  |  |  |  |  |  |  |  |  |  |
| **Q4015x** | 15º |  |  |  |  |  |  |  |  |  |  |  |  |

Número do Paciente:

**5000. PERDA DE PRODUTIVIDADE (do paciente e do responsável)**

| **Q5001** | Quantos membros vivem na casa? (Incluindo o paciente) |  |
| --- | --- | --- |
| **Q5002** | Quantas pessoas menores de 18 anos vivem em sua casa? (Incluindo o paciente) |  |
| **Q5003** | Quantas pessoas têm algum tipo de renda? |  |
| **Q5004** | Qual a renda total da família no último mês? | R$ |

| **Grau de parentesco com o paciente** |
| --- |
| 1. Pai/ mãe 2. Irmão/ Irmã 3. Filho/ Filha 4. Tio/ Tia 5. Avô/ Avó 6. Primo/ Prima 7. Cônjuge/ Companheiro (a) 8. Outros |

| **Escolaridade máxima alcançada** |
| --- |
| 1. Analfabeto/ Fundamental 1 incompleto 2. Fundamental 1 completo/ Fundamental 2 incompleto 3. Fundamental 2 completo/ Médio incompleto 4. Médio completo/ Técnico/ Superior incompleto 5. Superior completo |

| **Motivos de ausência** | |
| --- | --- |
| 1. Cuidando do paciente 2. Substituição do paciente ou do assistente 3. Enfermidade 4. Outros | |
| **Sexo** | |
| 1. Feminino | 1. Masculino |

| **Para respostas sim/não** | S. Sim | N. Não |
| --- | --- | --- |

ATENÇÃO: Completar com informações APENAS das pessoas ligadas ao paciente que o ajudaram nos cuidados durante a fase de doença.

|  | **Número do membro da família (a)** | **Código da relação com o paciente (b)** | **Código do sexo (c)** | **Idade em anos (d)** | **Escolaridade mais alta (e)** | **Estudando (S/N) (f)** | **Dias de ausência da escola (g)** | **Emprego remunerado (S/N) (h)** | **Dias de ausência do trabalho (i)** | **Código do motivo maior da ausência (j)** | **Renda total no último mês (k)** | **CUIDADO COM O PACIENTE** | |
| --- | --- | --- | --- | --- | --- | --- | --- | --- | --- | --- | --- | --- | --- |
|  |  |  |  |  |  |  |  |  |  |  |  | **Média diárias em horas (l)** | **Número de dias (m)** |
| **Q5005** | Paciente |  |  |  |  |  |  |  |  |  |  |  |  |
| **Q5006** | 1º |  |  |  |  |  |  |  |  |  |  |  |  |
| **Q5007** | 2º |  |  |  |  |  |  |  |  |  |  |  |  |
| **Q5008** | 3º |  |  |  |  |  |  |  |  |  |  |  |  |
| **Q5009** | 4º |  |  |  |  |  |  |  |  |  |  |  |  |
| **Q5010** | 5º |  |  |  |  |  |  |  |  |  |  |  |  |
| **Q5011** | 6º |  |  |  |  |  |  |  |  |  |  |  |  |
| **Q5012** | 7º |  |  |  |  |  |  |  |  |  |  |  |  |
| **Q5013** | 8º |  |  |  |  |  |  |  |  |  |  |  |  |
| **Q5014** | 9º |  |  |  |  |  |  |  |  |  |  |  |  |

**Formulário D - PACIENTES HOSPITALIZADOS**

**Coleta de dados do prontuário médico do paciente**

**Identificação do paciente:**

| **QM001** | Nome/Sobrenome | |  | | | | | | | | |
| --- | --- | --- | --- | --- | --- | --- | --- | --- | --- | --- | --- |
| **QM002** | Qual o número do registro médico ou número de identificação neste estabelecimento? | | | | | | |  | | | |
| **QM003** | Data da admissão | | */ /* | | | | | | | | |
| **QM004** | Diagnóstico da admissão hospitalar | 1. Diagnóstico não menciona dengue | | 1. Dengue sem classificação | 1. Dengue clássica | 1. Dengue clássica com manifestações hemorrágicas | 1. Febre hemorrágica do dengue | | 1. Síndrome de choque do dengue | 1. Outro | 1. Não registrado |

**Se o diagnóstico na admissão não menciona "dengue", por favor refira o(s) diagnóstico(s) da admissão. Do contrário vá para a questão QM006.**

| **QM005** | Especificar outros diagnósticos na admissão: | |  | | | | | | | | | |
| --- | --- | --- | --- | --- | --- | --- | --- | --- | --- | --- | --- | --- |
| **QM006** | Data da alta | | */ /* | | | | | | | | | |
| **QM007** | Diagnóstico da alta hospitalar | 1. Diagnóstico não menciona dengue | | 1. Dengue sem classificação | | 1. Dengue clássica | 1. Dengue clássica com manifestações hemorrágicas | 1. Febre hemorrágica do dengue | | 1. Síndrome de choque do dengue | 1. Outro | 1. Não registrado |
| **QM008** | O paciente contraiu alguma infecção hospitalar durante sua hospitalização? | | 1. Sim | | 1. Não | | | b. Se sim, qual? | | | | |
| **QM009** | O paciente foi hospitalizado anteriormente devido a esta enfermidade aguda? | | 1. Sim | | 1. Não | | | | 1. Não registrado | | | |
| **QM010** | O paciente teve alta com vida? | | 1. Sim | | 1. Não | | | | | | | |

**Sintomas**

| **QM011** | Data do início dos sintomas | */ /* | | | |
| --- | --- | --- | --- | --- | --- |
| **QM012** | Por quantos dias o paciente teve febre decorrente deste episódio de enfermidade? | | |  | |
| **QM013** | Dor de cabeça? | 1. Sim | 1. Não | | 1. Não registrado |
| **QM014** | Dor retrorbital? | 1. Sim | 1. Não | | 1. Não registrado |
| **QM015** | Mialgia/Artralgia? | 1. Sim | 1. Não | | 1. Não registrado |
| **QM016** | Anorexia? | 1. Sim | 1. Não | | 1. Não registrado |
| **QM017** | Exantema? | 1. Sim | 1. Não | | 1. Não registrado |
| **QM018** | Prurido? | 1. Sim | 1. Não | | 1. Não registrado |
| **QM019** | Vômitos? | 1. Sim | 1. Não | | 1. Não registrado |
| **QM020** | Dor epigástrica / abdominal? | 1. Sim | 1. Não | | 1. Não registrado |
| **QM021** | Sede excessiva? | 1. Sim | 1. Não | | 1. Não registrado |
| **QM022** | Manifestações hemorrágicas (petéquias, púrpura, equimoses, epistaxe, sangramento gengival, hematêmese/melena) | 1. Sim | 1. Não | | 1. Não registrado |
| **QM023** | Extravasamento plasmático (derrame pleural, derrame pericárdico, distensão abdominal, hepatomegalia, ascite) | 1. Sim | 1. Não | | 1. Não registrado |
| **QM024** | Diminuição da perfusão (diminuição da pressão arterial, convergência da pressão arterial, diminuição volume urinário, extremidades frias, pele fria e úmida, letargia, inquietude) | 1. Sim | 1. Não | | 1. Não registrado |

**Outros sinais**

**(Por favor, mencione outras manifestaçõesclínicas importantes que o paciente apresentou. Se não houver, vá para a QM030)**

| **QM025** | Manifestação clínica 1: |  | | |
| --- | --- | --- | --- | --- |
| **QM026** | Manifestação clínica 2: |  | | |
| **QM027** | Manifestação clínica 3: |  | | |
| **QM028** | Prova do Laço | 1. Positiva | 1. Negativa | 1. Não realizada |

**Exames Laboratoriais**

**(O paciente foi submetido a algum exame listado abaixo?)**

| **QM029** | Sorologia? | 1. Sim | 1. Não | | | | 1. Não registrado | | | Número de exames: | | | |
| --- | --- | --- | --- | --- | --- | --- | --- | --- | --- | --- | --- | --- | --- |
| **QM030** | Qual o resultado da sorologia? | | | | | | | | | | | | |
|  | Resultado 1, Qual tipo de exame? | 1. Reagente | | | | 1. Não reagente | | | | | 1. Inconclusivo | | |
|  | Resultado 2, Qual tipo de exame? | 1. Reagente | | | | 1. Não reagente | | | | | 1. Inconclusivo | | |
|  | Resultado 3, Qual tipo de exame? | 1. Reagente | | | | 1. Não reagente | | | | | 1. Inconclusivo | | |
|  | Resultado 4, Qual tipo de exame? | 1. Reagente | | | | 1. Não reagente | | | | | 1. Inconclusivo | | |
|  | Resultado 5, Qual tipo de exame? | 1. Reagente | | | | 1. Não reagente | | | | | 1. Inconclusivo | | |
| **QM031** | Pesquisa de antígeno NS1 | 1. Sim | | 1. Não | | | | 1. Não registrado | | | | | Número de exames: |
| **QM032** | Qual o resultado do NS1? | 1. Positivo | | | | 1. Negativo | | | | | 1. Inconclusivo | | |
| **QM033** | Isolamento viral? | 1. Sim | | 1. Não | | | | 1. Não registrado | | | | | Número de exames: |
| **QM034** | Qual o resultado do Isolamento viral? | 1. Positivo | | | | 1. Negativo | | | | | 1. Inconclusivo | | |
| **QM035** | Pesquisa de genoma do vírus dengue (RT-PCR) | 1. Sim | 1. Não | | 1. Não sabe | | | | Número de exames: | | | Resultados: | |
| **QM036** | Contagem de Plaquetas? | 1. Sim | | 1. Não | | | | 1. Não registrado | | | | | Número de exames: |
| **QM038** | Hematócrito? | 1. Sim | | | | 1. Não | | | | | 1. Não registrado | | |
| **QM039** | Quantos exames de hematócrito? |  | | | | | | | | | | | |
| **QM040** | Quantos hemogramas completos? |  | | | | | | | | | | | |
| **QM041** | Dosagem de albumina? | 1. Sim | | 1. Não | | | | 1. Não registrado | | | | | Número de exames: |
| **QM042** | Dosagem de ureia? | 1. Sim | | 1. Não | | | | 1. Não registrado | | | | | Número de exames: |
| **QM043** | Dosagem de Cálcio? | 1. Sim | | 1. Não | | | | 1. Não registrado | | | | | Número de exames: |
| **QM044** | Dosagem de Potássio? | 1. Sim | | 1. Não | | | | 1. Não registrado | | | | | Número de exames: |
| **QM045** | Dosagem de Sódio? | 1. Sim | | 1. Não | | | | 1. Não registrado | | | | | Número de exames: |
| **QM046** | Dosagem de Cloro? | 1. Sim | | 1. Não | | | | 1. Não registrado | | | | | Número de exames: |
| **QM047** | Dosagem de Glicemia? | 1. Sim | | 1. Não | | | | 1. Não registrado | | | | | Número de exames: |
| **QM048** | Dosagem de Creatinina? | 1. Sim | | 1. Não | | | | 1. Não registrado | | | | | Número de exames: |
| **QM049** | Velocidade de Hemossedimentação? | 1. Sim | | 1. Não | | | | 1. Não registrado | | | | | Número de exames: |
| **QM050** | Gasometria? | 1. Sim | | 1. Não | | | | 1. Não registrado | | | | | Número de exames: |
| **QM051** | Sumário de urina? | 1. Sim | | 1. Não | | | | 1. Não registrado | | | | | Número de exames: |
| **QM052** | Realizou outro(s) exame(s)? | 1. Sim | | | | 1. Não | | | | | 1. Não registrado | | |
| **QM053** | Qual exame 1? |  | | | | | | | | | | | |
| **QM054** | Quantas vezes? |  | | | | | | | | | | | |
| **QM055** | Qual exame 2? |  | | | | | | | | | | | |
| **QM056** | Quantas vezes? |  | | | | | | | | | | | |
| **QM057** | Qual exame 3? |  | | | | | | | | | | | |
| **QM058** | Quantas vezes? |  | | | | | | | | | | | |
| **QM059** | Qual exame 4? |  | | | | | | | | | | | |
| **QM060** | Quantas vezes? |  | | | | | | | | | | | |
| **QM061** | Qual exame 5? |  | | | | | | | | | | | |
| **QM062** | Quantas vezes? |  | | | | | | | | | | | |

**Exames de Imagem**

**(O paciente foi submetido a algum exame listado abaixo?)**

| **QM063** | Radiografia? | 1. Sim | 1. Não | Região do corpo: | Número de exames: |
| --- | --- | --- | --- | --- | --- |
| **QM064** | Ultrassonografia? | 1. Sim | 1. Não | Região do corpo: | Número de exames: |
| **QM065** | Tomografia computadorizada? | 1. Sim | 1. Não | Região do corpo: | Número de exames: |
| **QM066** | Ressonância nuclear magnética? | 1. Sim | 1. Não | Região do corpo: | Número de exames: |
| **QM067** | Realizou outro(s) exame(s) de imagem (ns)? | 1. Sim | 1. Não | Região do corpo: | Número de exames: |
| **QM068** | Qual(is) exame(s) de imagem(ns)? |  | | | |

**Procedimentos**

**(O paciente foi submetido a algum procedimento listado abaixo?)**

| **QM069** | Cirurgia | 1. Sim | 1. Não | | 1. Qual? |
| --- | --- | --- | --- | --- | --- |
| **QM070** | Cateterização Vesical | 1. Sim | 1. Não | | Quantas? |
| **QM071** | Cateterização Nasogástrica | 1. Sim | 1. Não | | Quantas? |
| **QM072** | Cateterização Venosa | 1. Sim | 1. Não | | Quantas? |
| **QM073** | Intubação Orotraqueal | 1. Sim | 1. Não | | Quantas? |
| **QM074** | Punção torácica | 1. Sim | 1. Não | | Quantas? |
| **QM075** | Outros procedimentos? | 1. Sim | | 1. Não | |
| **QM076** | Qual procedimento 1? |  | | | |
| **QM077** | Quantas vezes? |  | | | |
| **QM078** | Qual procedimento 2? |  | | | |
| **QM079** | Quantas vezes? |  | | | |
| **QM080** | Qual procedimento 3? |  | | | |
| **QM081** | Quantas vezes? |  | | | |
| **QM082** | Qual procedimento 4? |  | | | |
| **QM083** | Quantas vezes? |  | | | |
| **QM084** | Qual procedimento 5? |  | | | |
| **QM085** | Quantas vezes? |  | | | |

**Material e Medicamentos**

**(O paciente utilizou algum material ou medicamento listado abaixo?)**

| **QM086** | Unidades de soro fisiológico | 1. Sim | | | 1. Não | | | Quantidade: | |
| --- | --- | --- | --- | --- | --- | --- | --- | --- | --- |
| **QM087** | Unidades de solução glicosada | 1. Sim | | | 1. Não | | | Quantidade: | |
| **QM088** | Unidades de solução de Ringer Lactato | 1. Sim | | | 1. Não | | | Quantidade: | |
| **QM089** | Expansores plasmáticos (dextran, gelatina) | 1. Sim | | | 1. Não | | | Quantidade: | |
| **QM090** | Unidades de albumina | 1. Sim | | | 1. Não | | | Quantidade: | |
| **QM091** | Concentrado de hemácias | 1. Sim | | | 1. Não | | | Quantidade: | |
| **QM092** | Concentrado de plaquetas | 1. Sim | | | 1. Não | | | Quantidade: | |
| **QM093** | Potássio | 1. Sim | 1. Não | Substância (nome genérico): | | Apresentação: | Concentração: | | Número de doses utilizadas: |
| **QM094** | Sódio | 1. Sim | 1. Não | Substância (nome genérico): | | Apresentação: | Concentração: | | Número de doses utilizadas: |
| **QM095** | Bloqueadores H2 ou inibidores da bomba de prótons | 1. Sim | 1. Não | Substância (nome genérico): | | Apresentação: | Concentração: | | Número de doses utilizadas: |
| **QM096** | Analgésicos/antitérmicos durante a internação | 1. Sim | 1. Não | Substância (nome genérico): | | Apresentação: | Concentração: | | Número de doses utilizadas: |
| **QM097** | Antiemético durante a internação | 1. Sim | 1. Não | Substância (nome genérico): | | Apresentação: | Concentração: | | Número de doses utilizadas: |
| **QM098** | Antibiótico durante a internação | 1. Sim | 1. Não | Substância (nome genérico): | | Apresentação: | Concentração: | | Número de doses utilizadas: |
| **QM099** | Outro medicamento durante a internação | 1. Sim | 1. Não | Substância (nome genérico): | | Apresentação: | Concentração: | | Número de doses utilizadas; |
| **QM100** | Sonda Nasogástrica | 1. Sim | | | 1. Não | | Quantidade: | | |
| **QM101** | Cateter Vesical | 1. Sim | | | 1. Não | | Quantidade: | | |

**Informações sobre a internação/Diárias hospitalares**

| **QM102** | Quantos dias de internação em enfermaria? |  |
| --- | --- | --- |
| **QM103** | Quantos dias de internação em quarto/ apartamento? |  |
| **QM104** | Quantos dias de internação em setor de emergência? |  |
| **QM105** | Quantos dias o paciente esteve internado em uma unidade de terapia intensiva (UTI)? |  |

**Honorários Profissionais (Preencher para hospitais da rede privada)**

| **QM106** | Durante a internação quantas visitas feitas por médicos? |  |
| --- | --- | --- |
