# Supplementary material for: Economic Impact of Dengue: Multicenter Study across Four Brazilian Regions
Source: PLoS Negl Trop Dis. 2015 Sep 24;9(9):e0004042. doi: 10.1371/journal.pntd.0004042 (PMC4581827; doi:10.1371/journal.pntd.0004042)
Supplement: S2 Text — (DOCX) [file pntd.0004042.s004.docx]

**S2 Text.** Assumptions to estimate cost per dengue case using primary data from six cities and extrapolating to national level

*Calculating the costs per case primary data (Table 3 [see main text]):*

1. Ambulatory cases, cost components

**Cost_Total_ambulatory_ = Cost_Direct_ambulatory_ + Cost_Indirect_ambulatory_**

where

**Cost_Direct_ambulatory_ = Cost_Medicalvisit + Cost_Medication + Cost_ Labexams (**Exams**) + Cost_NMDirect (**food, lodging and transportation**)**

and

**Cost_Indirect_ambulatory =_** Cost of school days and/or work lost by patient and/or caregivers

2. Hospitalized cases, cost components

Includes:

Cost of hospital stay, medication, laboratory/exams and non-medical costs

**Cost_Total_hospital_ = Cost_Direct_hospital_ + Cost_Indirect_hospital_**

where

**Cost_Direct_hospital_ = Cost_Medicalvisit + Cost_Medication +**

**Cost_ Labexams + Cost_NMDirect + Cost_Direct_ambulatory_ X %(hospitalized patients consuming ambulatory care)**

and

**Cost_Indirect_hospital =_** Cost of school days and/or work lost by patient and/or caregivers

*Estimating the number of cases in each city:*

Given that reported cases (SINAN - Table 1 [see main text]) do not discriminate into ambulatory versus hospital cases nor between public versus private, we made the following assumptions:

1. Number of reported cases in the six cities (**RCase_sinan_**): data from SINAN (Table 1 [see main text])
2. We adopted the ratio of ambulatory:hospital (9:1) to assess the number of ambulatory cases and hospitalized cases reported by SINAN

**RCase_ambulatory_ = RCase_Sinan_ *X* 90%**

**RCase_hospital_ = RCase_Sinan_ *X* 10%**

1. We weighted the number of cases by public and private sector attendance according to the data census (IBGE, 2012). In the Southeast and Midwest regions, 65% of the patients attended the public sector; in the North and Northeast regions, 75% of the patients attended the public sector. This assumption was for both outpatients and inpatients.

**RCase_ambulatory public_ = RCase_ambulatory_ *X* 65% (Southeast and Midwest)**

**RCase_ambulatory public_ = RCase_ambulatory_ *X* 75% (North and Northeast)**

**RCase_ambulatory private_= RCase_ambulatory_ *X* 35% (Southeast and Midwest)**

**RCase_ambulatory private_= RCase_ambulatory_ *X* 25% (North and Northeast)**

Similarly we applied the above equations for hospitalized cases.

The tables below show the estimated number of outpatients and inpatients according to the 6 cities.

Table S2a. Estimated number of outpatient dengue cases by site using SINAN data (September 2012 – August 2013)

| Cases | Midwest | Southeast | | Northeast | | North | Overall |
| --- | --- | --- | --- | --- | --- | --- | --- |
|  | Goiania | Belo Horizonte | Rio de Janeiro | Teresina | Recife | Belem |  |
| Public | 35,144 | 77,579 | 43,358 | 1,818 | 2,749 | 1,190 | 161,838 |
| Private | 18,924 | 41,773 | 23,347 | 606 | 916 | 397 | 85,963 |
| Total | 54,068 | 119,353 | 66,704 | 2,425 | 3,666 | 1,587 | 247,801 |

Table S2b. Estimated number of inpatient dengue cases by site using SINAN data (September 2012 – August 2013)

| Cases | Midwest | Southeast | | Northeast | | North | Overall |
| --- | --- | --- | --- | --- | --- | --- | --- |
|  | Goiania | Belo Horizonte | Rio de Janeiro | Teresina | Recife | Belem |  |
| Public | 3,905 | 8,620 | 4,818 | 202 | 305 | 132 | 17,982 |
| Private | 2,103 | 4,641 | 2,594 | 67 | 102 | 44 | 9,551 |
| Total | 6,008 | 13,261 | 7,412 | 269 | 407 | 176 | 27,533 |

Therefore,

Reported cases = ∑ estimated number of ambulatory and hospitalized cases (Tables S2a and S2b)

Example for the city of Goiania regarding the number of patients in ambulatory or hospital settings: 54,068 outpatients + 6,008 inpatients = 60,076 reported cases in this city (Table 1 (see main text), line 2, column 2).

Regarding the number of patients split by public or private sectors we applied the total of ambulatory cases 54,068 * 0.65 = 35,144 cases in the public sector (Table S2a).

*Calculating the total cost of ambulatory and hospitalized cases – primary data (Tables S2c and S2d).*

The cost of ambulatory cases in the selected cities is the estimated number of outpatients (SINAN, Table S2a) multiplied by the cost per case according to cost component and public and private sector (Table 3 [see main text]). Similarly, we estimated the total cost for hospitalized cases.

Given the cost variations between the 6 cities, we estimated a cost per case taking into consideration the primary data derived from each city and the registered cases.

First, we calculate the Grand total cost for ambulatory cases considering the 6 cities together as: $42,952,711 (Table S2c). Second, we divided this cost by the number of ambulatory cases (Table S2a). Therefore, the estimated cost per ambulatory case (societal perspective) was $ 42,952,711/247,801 ambulatory cases = $173 (Table 4 [see main text]).

Similarly, we estimated the cost per hospitalized cases.

*Estimating the costs of dengue in Brazil:*

1. We retrieved national data from the number of reported cases (**RNCase_Sinan_=** 2,013,274**)** by the official notification system (SINAN), during the study period (Table 1 [see main text]).
2. We also adopted the ratio of ambulatory:hospital (9:1) as a parameter to estimate the number of hospitalized and ambulatory cases at national levels.

We applied two expansion factor for the national reported cases in order to adjust for underreporting.

**RNCase_ambulatory_ = RNCase_Sinan_ *X* 90% *X* 2**

**RNCase_hospital_ = RNCase_Sinan_ *X* 10% *X* 1.6**

1. At national level, the total annual economic burden (EB_total_) was estimated as the reported national cases (SINAN) (EFs) multiplied by the estimated cost per cases. These results were stratified by ambulatory and hospital cases.

**EB_totalambulatory_= EB_cities_X RNCase_ambulatory_**

**EB_totalhospital_= EB_cities_ X RNCase_hospital_**

**Table S2c.** Cost of ambulatory dengue cases in selected cities based on the registered cases (SINAN), stratified by health sector (private or public),^#^ considering societal perspective (2013 US$)

| Cost component, US$ | | Midwest | Southeast | | Northeast | | North | Overall |
| --- | --- | --- | --- | --- | --- | --- | --- | --- |
|  |  | Goiania | Belo Horizonte | Rio de Janeiro | Teresina | Recife | Belem |  |
| Direct costs |  |  |  |  |  |  |  |  |
| Public |  | 1,941,193 | 6,939,404 | 1,325,784 | 59,268 | 108,123 | 44,670 | 10,418,441 |
| Private |  | 3,172,610 | 6,646,768 | 2,118,531 | 80,542 | 70,626 | 0 | 12,089,077 |
| Subtotal direct costs |  | 5,113,803 | 13,586,172 | 3,444,314 | 139,810 | 178,749 | 44,670 | 22,507,518 |
| Indirect costs |  |  |  |  |  |  |  |  |
| Public |  | 3,588,519 | 4,512,168 | 1,368,785 | 71,209 | 167,643 | 33,665 | 9,741,989 |
| Private |  | 3,128,233 | 4,450,635 | 2,787,145 | 222,622 | 114,568 | 0 | 10,703,204 |
| Subtotal indirect costs |  | 6,716,752 | 8,962,804 | 4,155,930 | 293,831 | 282,211 | 33,665 | 20,445,193 |
| Total |  |  |  |  |  |  |  |  |
| Public |  | 5,529,712 | 11,451,572 | 2,694,569 | 130,477 | 275,766 | 78,335 | 20,160,430 |
| Private |  | 6,300,843 | 11,097,404 | 4,905,676 | 303,164 | 185,194 | 0 | 22,792,281 |
| Grand total costs |  | 11,830,555 | 22,548,976 | 7,600,245 | 433,641 | 460,960 | 78,335 | 42,952,711 |

^#^We assumed that 65% of patients attended the public sector in the Southeast and Midwest regions and 75% in the North and Northeast regions (Instituto Brasileiro de Geografia e Estatistica. Pesquisa Mensal de Emprego, 2013. IBGE. 2013:32).

**Table S2d.** Cost of hospitalized dengue cases in selected cities based on the registered cases (SINAN), stratified by health sector (private or public), ^#^ considering societal perspective (2013 US$)

| Cost component, US$ | | Midwest | Southeast | | Northeast | | North | Overall |
| --- | --- | --- | --- | --- | --- | --- | --- | --- |
|  |  | Goiania | Belo Horizonte | Rio de Janeiro | Teresina | Recife | Belem |  |
| Direct costs |  | |  |  |  |  |  |  |
| Public | 885,498 | | 3,240,877 | 0 | 39,930 | 64,783 | 31,376 | 4,262,464 |
| Private | 945,762 | | 2,206,806 | 2,351,272 | 44,965 | 69,027 | 14,003 | 5,631,835 |
| Subtotal direct costs | 1,831,260 | | 5,447,683 | 2,351,272 | 84,895 | 133,810 | 45,379 | 9,894,299 |
| Indirect costs |  | |  |  |  |  |  |  |
| Public | 667,962 | | 887,817 | 0 | 14,475 | 15,871 | 0 | 1,586,125 |
| Private | 249,905 | | 36,0167 | 144,470 | 10,689 | 91,786 | 0 | 857,016 |
| Subtotal indirect costs | 917,867 | | 1,247,984 | 144,470 | 25,164 | 107,656 | 0 | 2,443,141 |
| Total |  | |  |  |  |  |  |  |
| Public | 1,553,460 | | 4,128,694 | 0 | 54,405 | 80,654 | 31,376 | 5,848,588 |
| Private | 1,195,667 | | 2,566,973 | 2,495,742 | 55,654 | 160,813 | 14,003 | 6,488,852 |
| Grand total costs | 2,749,127 | | 6,695,667 | 2,495,742 | 110,059 | 241,467 | 45,379 | 12,337,440 |

^#^ We assumed that 65% of patients attended the public sector in the Southeast and Midwest regions and 75% in the North and Northeast regions (Instituto Brasileiro de Geografia e Estatistica. Pesquisa Mensal de Emprego, 2013. IBGE. 2013:32).
